# Central memory T cells with key TCR repertoires and gene expression profiles dominate influenza CD8+ T cell pools across the human lifespan

**DOI:** 10.1073/pnas.2501167122

**Published:** 2025-07-22

**Authors:** Tejas Menon, Hayley A. McQuilten, Jerome Samir, Thi H. O. Nguyen, Ratana Lim, Jasveen Kaur, Simone Rizzetto, Auda Eltahla, Paul G. Thomas, Martha Lappas, Jamie Rossjohn, Stephanie Gras, Jane Crowe, Katie L. Flanagan, Fabio Luciani, Peter C. Doherty, Carolien E. van de Sandt, Katherine Kedzierska

**Affiliations:** ^a^Department of Microbiology and Immunology, University of Melbourne, Peter Doherty Institute, Parkville, VIC 3000, Australia; ^b^School of Biomedical Sciences, University of New South Wales, Sydney, NSW 2052, Australia; ^c^Department of Obstetrics and Gynaecology, University of Melbourne, Melbourne, VIC 3000, Australia; ^d^School of Health Sciences and School of Medicine, University of Tasmania, Launceston, TAS 7248, Australia; ^e^Department of Host-Microbe Interactions, St. Jude Children’s Research Hospital, Memphis, TN 38105; ^f^Immunity Program and Department of Biochemistry and Molecular Biology, Biomedicine Discovery Institute, Monash University, Clayton, VIC 3800, Australia; ^g^Institute of Infection and Immunity, Cardiff University School of Medicine, Cardiff CF14 4XN, United Kingdom; ^h^Viral and Structural Immunology Laboratory, Department of Biochemistry and Chemistry, La Trobe Institute for Molecular Science, La Trobe University, Bundoora, VIC 3083, Australia; ^i^Deepdene Surgery, Deepdene, VIC 3103, Australia; ^j^School of Health and Biomedical Science, Royal Melbourne Institute of Technology, Melbourne, VIC 3000, Australia; ^k^Tasmanian Vaccine Trial Centre, Clifford Craig Foundation, Launceston General Hospital, Launceston, TAS 7248, Australia

**Keywords:** memory T cells, central memory, influenza-specific T cells, T cell receptors, human lifespan

## Abstract

Central memory CD8^+^ T cells (T_cm_) represent the prominent memory T cell subset in human blood, yet the persistence of T cell receptor (TCR) clonotypic and transcriptional features of epitope-specific T_cm_ pools across the human lifespan remains unknown. We analyzed T_cm_ CD8^+^ T cells specific for HLA-A*02:01-M1_58–66_ (A2/M1_58_; a prominent influenza epitope) in newborns, children, adults, and older adults directly ex vivo. Our data provide evidence that epitope-specific T_cm_ CD8^+^ pools dominate influenza-specific memory A2/M1_58_^+^CD8^+^ T cell responses from the early childhood until old age. T_cm_ gene signatures were largely maintained across the age groups, although self-renewal genes defined T_cm_ pools in children, while older adult T_cm_ A2/M1_58_^+^CD8^+^ T cells displayed detoxication and stress profiles. TCRαβ diversity within T_cm_ A2/M1_58_^+^CD8^+^ T cell pools was greater in children and older adults, when compared to adults. The key public-associated TCRαβ clonotypes largely persisted across the human lifespan, although their highest frequency was detected in adults, reflecting lower TCRαβ diversity in this group. Older adults displayed increased TCRαβ heterogeneity, underpinned by large TCRαβ clonotype expansions of private TCRαβ clonotypes. Our study highlights the importance of largely preserved virus-specific T_cm_ pools across the human lifespan and advocates for boosting persistent TCRαβ clonotypes within this key peripheral blood subset.

## Significance

Central memory CD8^+^ T cells are important in providing protection against viral infections, including influenza. However, it is not well understood how the transcriptomic features and T cell receptor (TCR) repertoires of influenza-specific central memory CD8^+^ T cells change across the human lifespan. We studied central memory CD8^+^ T cells specific for the prominent and conserved influenza A derived HLA-A*02:01-restricted M1_58–66_ peptide (A2/M1_58_). We show that gene signatures of central memory A2/M1_58_^+^ CD8^+^ T cells are largely similar across the human lifespan and key TCRs are maintained within the central memory A2/M1_58_^+^ CD8^+^ pools. Our study highlights persistence of the central memory CD8^+^ T cell subset across the human lifespan and advocates for boosting persistent TCRs within this subset.

Long-lasting memory CD8^+^ T cells provide rapid recall responses toward evolving viruses, including influenza viruses and SARS-CoV-2, thereby reducing disease severity (1–3). CD8^+^ T cells recognize peptides (p) bound to Human Leukocyte Antigen class I (HLA-I) on virus-infected cells via their T cell receptors (TCRs). Following TCR activation, T cells expand and differentiate into effector CD8^+^ T cells (4). Following viral clearance, ~5 to 10% of T cells establish long-lasting memory pools. Epitope-specific CD8^+^ T cells are generally studied in adults, yet how virus-specific memory T cell pools evolve across the human lifespan remains elusive.

Circulating memory CD8^+^ T cells consist of stem cell memory (T_scm_), central memory (T_cm_), effector memory (T_em_), and terminally differentiated (T_emra_) memory (5–7), with their own distinct circulation patterns, proliferative capacity, and functionality. Self-renewing multipotent T_scm_ cells reside in the circulation and lymph nodes and generate T_cm_ and T_em_ cells (7, 8). They are important in chronic infections to replenish exhausted or senescent effector T cells (9). T_em_ and T_emra_ cells occupy the circulation and tissues, where T_em_ cells have potent and rapid effector functions and T_emra_ cells represent the end-stage of memory T cell differentiation, with less cytokine production, proliferative ability, and cytotoxic ability (5, 6, 10). T_emra_ cells are associated with chronic infections, including cytomegalovirus (CMV) (10, 11). T_cm_ cells are vital, as they maintain long-lived and self-renewing properties, contribute to recall responses along with T_em_ cells, and generate tissue-resident memory populations (12–14). CD8^+^ T cells specific for influenza A virus (IAV) (15, 16), influenza B virus (IBV) (15, 17), and SARS-CoV-2 (18–20) are predominantly T_cm_.

The lineage relationship between T cell memory subsets is the subject of ongoing discussion, with different models proposed. The decreasing potential model states that TCR stimulation drives T cells to differentiate through each subset, with memory potential decreasing and effector differentiation increasing (T_naive_→T_scm_→T_cm_→T_em_→T_emra_) (6, 21, 22). The circular “on–off–on” model, where effector T cells redifferentiate into different memory T cell subsets and dedifferentiate into effector T cells upon rechallenge (14, 23). The asymmetric division model proposes that uneven distribution of important transcription factors and epigenetic regulators between daughter cells, lead to one daughter with high effector potential and the other displaying high memory potential (22, 24). Finally, the distinct lineage model argues that TCR clonotype determines the differentiation fate into a single memory subset (25). Recent work suggests that T cell lineage relationship may be explained by a mix of models (26).

Previous reports assessed effects of aging on memory composition of antigen-specific CD8^+^ T cell subsets. In bulk T cell populations, T_naive_ cells are abundant in newborns but decrease with age, while memory T cell populations increase. T_emra_ cells are ample in older adults, likely driven by chronic infections like CMV (11, 27). There is also an interest in the effect of aging on changes in the TCRαβ repertoire due to its importance for T cell functionality (16, 28–30). Studies revealed decreased TCRαβ diversity with increasing age, increases in clonal expansion and increased complementarity determining region-3 (CDR3)α length (16, 30–32). Analyses of paired TCRαβ from epitope-specific CD8^+^ T cells across age are rare (16, 30–32). Our recent analyses of CD8^+^ T cells specific for the prominent and conserved HLA-A*02:01-restricted M1_58–66_ peptide derived from IAV (A2/M1_58_) revealed that highly functional epitope-specific public TCRαβ clonotypes (shared between individuals) are gradually replaced with less functional private clonotypes (expanded but not shared between individuals) with age (16, 30).

Studies largely focused on bulk T cell populations or total epitope-specific CD8^+^ T cells. In-depth analysis on gene signatures and clonal TCR composition of antigen-specific memory subsets, especially prominent T_cm_, across the human lifespan has not been explored. Here, we defined how gene signatures and TCRαβ repertoire composition change within the influenza A2/M1_58_-specific CD8^+^ T cell memory compartments across the human lifespan. We established that T_cm_ A2/M1_58_^+^CD8^+^ pools became the most prominent memory population during early childhood and remained dominant across the human lifespan, including minimal changes within the gene profiles and TCRαβ repertoire composition. We provide insights in how virus epitope-specific CD8^+^ T cell memory populations are maintained across the human lifespan and emphasize the key importance of establishing long-lived virus-specific T_cm_ CD8^+^ T cell pools across the human lifespan.

## Results

### T_cm_ Pools Dominate Influenza-Specific Memory A2/M1_58_^+^CD8^+^ T Cell Responses from Early Childhood.

**Fig. 1.**
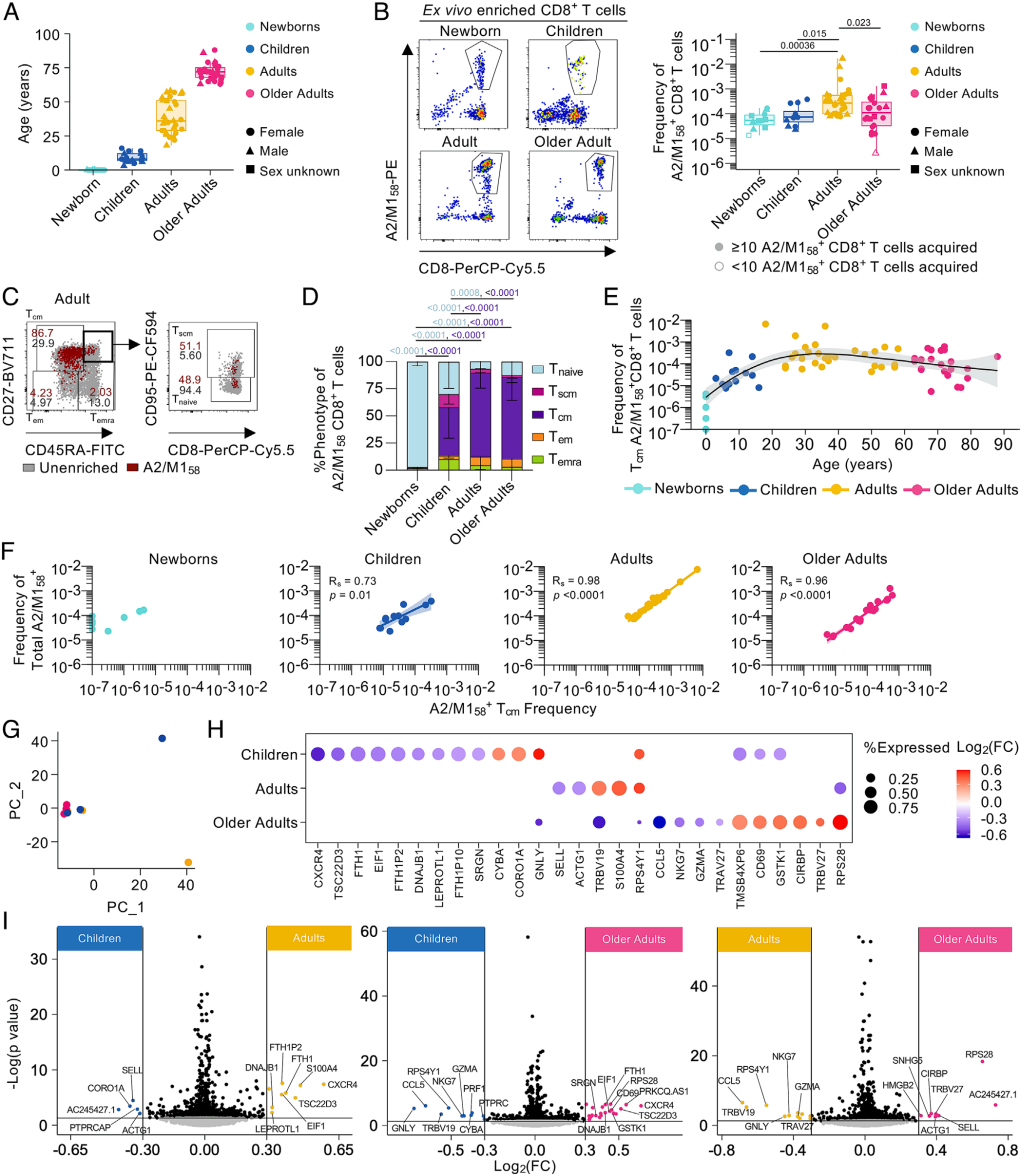
Central memory T cells dominate influenza-specific CD8^+^ T cells across the human lifespan. (*A*) Age of HLA-A*02:01-expressing newborns, children, adults, and older adults. (*B*) Representative FACS plots of enriched tetramer-specific A2/M1_58_^+^CD8^+^ T cells and frequencies of total A2/M1_58_^+^CD8^+^ T cells. (*C*) Representative FACS plots of A2/M1_58_^+^CD8^+^ T_cm_-like (CD27^+^CD45RA^−^) cells, T_em_-like (CD27^−^CD45RA^−^), T_emra_-like (CD27^-^CD45RA^+^), T_naive_-like (CD27^+^CD45RA^+^CD95^−^), T_scm_-like (CD27^+^CD45RA^+^CD95^+^) cells. Gray dots represent total CD8^+^ T cells in unenriched samples, red dots A2/M1_58_^+^CD8^+^ T cells in enriched samples. Gating across age groups shown in *SI Appendix*, Fig. S1*B*. (*D*) Stacked bar plots of memory phenotype proportion across age groups. Statistical significance was determined with two-way ANOVA with a two-sided Tukey’s test for multiple comparisons. (*E*) Frequency of T_cm_ A2/M1_58_^+^CD8^+^ T cells across age. (*F*) Correlation of the frequency of total A2/M1_58_^+^CD8^+^ T cells and frequency of A2/M1_58_^+^CD8^+^ T_cm_-like cells using Spearman’s rank correlation (R_s_) (n = 10; Newborns, n = 12; Children, n = 30; Adults, n = 22; Older Adults). (*G*) Principal Component Analysis of scRNASeq data of donors. Each dot represents a donor and is colored by age group. (*H*) Bubble plot of differentially expressed genes (DEG) from A2/M1_58_^+^CD8^+^ T_cm_ from children, adults, and older adults. DEGs were identified by pairwise comparison with a two-side hurdle model (MAST) without correction for multiple comparison (*P* < 0.05). (*I*) Pairwise volcano plots of DEGs in A2/M1_58_^+^CD8^+^ T_cm_ in children, adults, and older adults. Significant genes were those with |Log_2_(fold change)| >0.3 and *P*-value <0.05. (*A* and *C*) A2/M1_58_^+^CD8^+^ T_cm_ frequencies of 0 are plotted as 10^−7^. Donors with <10 total A2/M1_58_^+^CD8^+^ T cell counts were excluded from phenotypic analysis.

To define antigen-specific memory CD8^+^ T cells development across the human lifespan, we analyzed memory CD8^+^ T cells toward the prominent human A2/M1_58_ epitope, in HLA-A*02:01-expressing newborns (n = 11; 0-y; 27.3% female), children (n = 17; median 8-y, range 3 to 16; 52.9% female), adults (n = 33; median 36-y, range 18 to 58; 45.5% female), and older adults (n = 27; median 72-y, range 63 to 88; 63% female) (Fig. 1*A* and *SI Appendix*, Table S1).

To quantify the magnitude of A2/M1_58_^+^CD8^+^ T cells in blood across age, we performed tetramer-associated magnetic enrichment (TAME) on PBMCs (16). Tetramer-positive A2/M1_58_^+^CD8^+^ T cell frequencies increased with age from newborns (median 5.34 × 10^−5^; range 1.35 × 10^−5^−1.66 × 10^−4^), to children (7.43 × 10^−5^; 2.27 × 10^−5^-3.80 × 10^−4^), peaked in adults (2.66 × 10^−4^; 7.68 × 10^−5^-1.7 × 10^−2^), and declined in older adults (1.19 × 10^−4^; 2.56 × 10^−6^−1.30 × 10^−3^) (Fig. 1*B* and *SI Appendix*, Fig. S1*A*). We defined memory subsets within influenza-specific A2/M1_58_^+^CD8^+^ T cells across the human lifespan, namely T_naïve_ (CD27^+^CD45RA^+^CD95^-^), T_scm_ (CD27^+^CD45RA^+^CD95^+^), T_cm_ (CD27^+^CD45RA^-^), T_em_ (CD27^-^CD45RA^-^), and T_emra_ (CD27^-^CD45RA^+^) subsets, ex vivo (Fig. 1*C* and *SI Appendix*, Fig. S1*B*). T_naive,_ T_scm_, T_em_, and T_emra_ A2/M1_58_^+^CD8^+^ T cell pools were identified in children, adults, and older adults. While T_naive_ were most prevalent in newborns, T_cm_ influenza-specific A2/M1_58_^+^CD8^+^ T cells dominated from early childhood to older age, increased with age, peaked in adults, and were relatively well maintained in older adults (Fig. 1 *D* and *E* and *SI Appendix*, Fig. S1*C*). T_cm_ A2/M1_58_^+^CD8^+^ T cell frequencies strongly correlated with the frequency of total tetramer-positive A2/M1_58_^+^CD8^+^ T cells across age groups except newborns (children R_s_ = 0.73, *P* = 0.01; adults R_s_ = 0.98, *P* < 0.0001; older adults R_s_ = 0.96, *P* < 0.0001) (Fig. 1*F*). Similar trends were observed for other phenotypes (*SI Appendix*, Fig. S1*D*).

We demonstrated that conversely to the total A2/M1_58_^+^CD8^+^ T cell population declining with age, T_cm_ A2/M1_58_^+^CD8^+^ memory pools are well maintained across the human lifespan. We focused on transcriptomic and clonal TCR repertoire changes within the prominent T_cm_ subset across the human lifespan.

### Age-Specific T_cm_ A2/M1_58_^+^CD8^+^ T Cell Gene Signatures Between Age Groups.

Given the dominance of T_cm_ cells across the human lifespan, we identified age-related changes in the transcriptomic profiles of T_cm_ A2/M1_58_^+^CD8^+^ T cells across age groups via our single-cell RNA sequencing (scRNASeq) A2/M1_58_^+^CD8^+^ T cell dataset (16). As newborns had low frequencies of T_cm_ A2/M1_58_^+^CD8^+^ T cells (Fig. 1 *D*–*F*), we analyzed T_cm_ A2/M1_58_^+^CD8^+^ T cells in children (130 T_cm_ cells), adults (142 T_cm_ cells), and older adults (82 T_cm_ cells).

Principal Component Analysis revealed that T_cm_ A2/M1_58_^+^ CD8^+^ T cells from most donors had similar gene expression profiles across age; not surprising as all the cells in this dataset are CD8^+^ T cells specific for the same antigen (Fig. 1*G*). Differential gene expression analysis showed that children’s T_cm_ A2/M1_58_^+^CD8^+^ T cell gene signatures displayed reduced expression of *TSC22D3* [role in TCR signaling inhibition (33)], *EIF1* [translation initiation (34)] and *DNAJB1* [protein folding (35)], but higher expression of *CYBA* and *CORO1A*, which play a role in NADPH oxidase activity (needed for CD8^+^ T cell effector function (36, 37) and T cell survival (38) respectively). Children’s T_cm_ A2/M1_58_^+^CD8^+^ T cells displayed the lowest expression of the effector phenotype gene *CXCR4* (39), granzyme storage gene *SRGN* (40), and T cell activation gene *CD69*, while expression of cytotoxic gene *GNLY* was highest in children compared to adults and older adults (Fig. 1*H*).

Adult T_cm_ A2/M1_58_^+^CD8^+^ T cell gene expression profiles were characterized by high *TRBV19* expression [associated with highly functional A2/M1_58_^+^CD8^+^ T cells (16, 41, 42)] and reduced expression of *SELL* (Fig. 1*H*) consistent with its enrichment in total adult A2/M1_58_^+^CD8^+^ T cells (16).

T_cm_ A2/M1_58_^+^CD8^+^ T cell gene expression profiles in older adults displayed reduced expression of memory-associated gene *CCL5*, cytotoxic-associated genes *GNLY*, *GZMA,* and *NKG7* and public TCR-associated *TRBV19* and *TRAV27* genes, while the private TCR-associated *TRBV27* was enriched in the older adult gene expression profile (16) (Fig. 1*H*). Unique for older adult T_cm_ A2/M1_58_^+^CD8^+^ T cells, we observed increased expression of TCR activation-associated gene *CD69*, stress-induced gene *CIRBP* (43), ribosomal 40S component gene *RPS28* (44), and cellular detoxification gene *GSTK1* which is involved in the antioxidant glutathione function, important for T cell proliferation after activation (45, 46), (Fig. 1*H*). Gene set enrichment analysis also revealed enrichment of a “stress” signature in older adult T_cm_ A2/M1_58_^+^CD8^+^ T cells which was associated with the differentially expressed *DNAJB1* gene (*SI Appendix*, Fig. S2*A*). Differentially expressed *NKG7*, *GZMA*, and *PRF1* were associated with the higher “CD8 cytotoxic”, “CD8 EM”, “NK-like activating”, and “NK-like cytotoxicity” signatures in children and adults.

Pairwise comparison between children and adult T_cm_ A2/M1_58_^+^CD8^+^ T cells showed that children had higher expression of *SELL* and lower expression of *CXCR4,* indicating that children’s T_cm_ pools are less differentiated compared to those of adults (Fig. 1*I*). This may indicate that children’s T_cm_ A2/M1_58_^+^CD8^+^ T cells may maintain self-renewal qualities of naïve cells, as shown by increased expression of *CORO1A*. However, when comparing T_cm_ A2/M1_58_^+^CD8^+^ T cells from children and older adults, no differences in *SELL* and *CORO1A* were observed, while *CXCR4* expression was higher in older adult T_cm_ A2/M1_58_^+^CD8^+^ T cells (Fig. 1*I*). Conversely, cytotoxic-associated (*GNLY*, *PRF1*, *GZMA, NKG7*) and effector (*CCL5*) genes were highly expressed among children’s T_cm_ A2/M1_58_^+^CD8^+^ T cells compared to older adults. Similarly adult T_cm_ A2/M1_58_^+^CD8^+^ T cells displayed a prominent cytotoxic/effector profile compared to older adults (Fig. 1*I*). This trend of lower effector potential in older adult T_cm_ A2/M1_58_^+^CD8^+^ T cells was less clear on a protein level (Granzyme A, Granulysin, CD69, NKG7, and Perforin) directly ex vivo (*SI Appendix*, Fig. S2*B*). There was a trend (nonsignificant) for lower CXCR4 expression in children and lower TRBV19 expression in older adults (S*I Appendix*, Fig. S2*B*), consistent with scRNASeq data (Fig. 1*H*).

Overall, we observed that T_cm_ A2/M1_58_^+^CD8^+^ T cells in children expressed self-renewal properties, while T_cm_ A2/M1_58_^+^CD8^+^ T cells in older adults had greater cellular detoxication and stress-induced genes. A strong cytotoxic/effector gene expression profile in child and adult T_cm_ A2/M1_58_^+^CD8^+^ T cells but was not reflected in protein expression of resting cells directly ex vivo.

### Greater TCRαβ Diversity within T_cm_ A2/M1_58_^+^CD8^+^ T Cell Pools in Children and Older Adults.

**Fig. 2.**
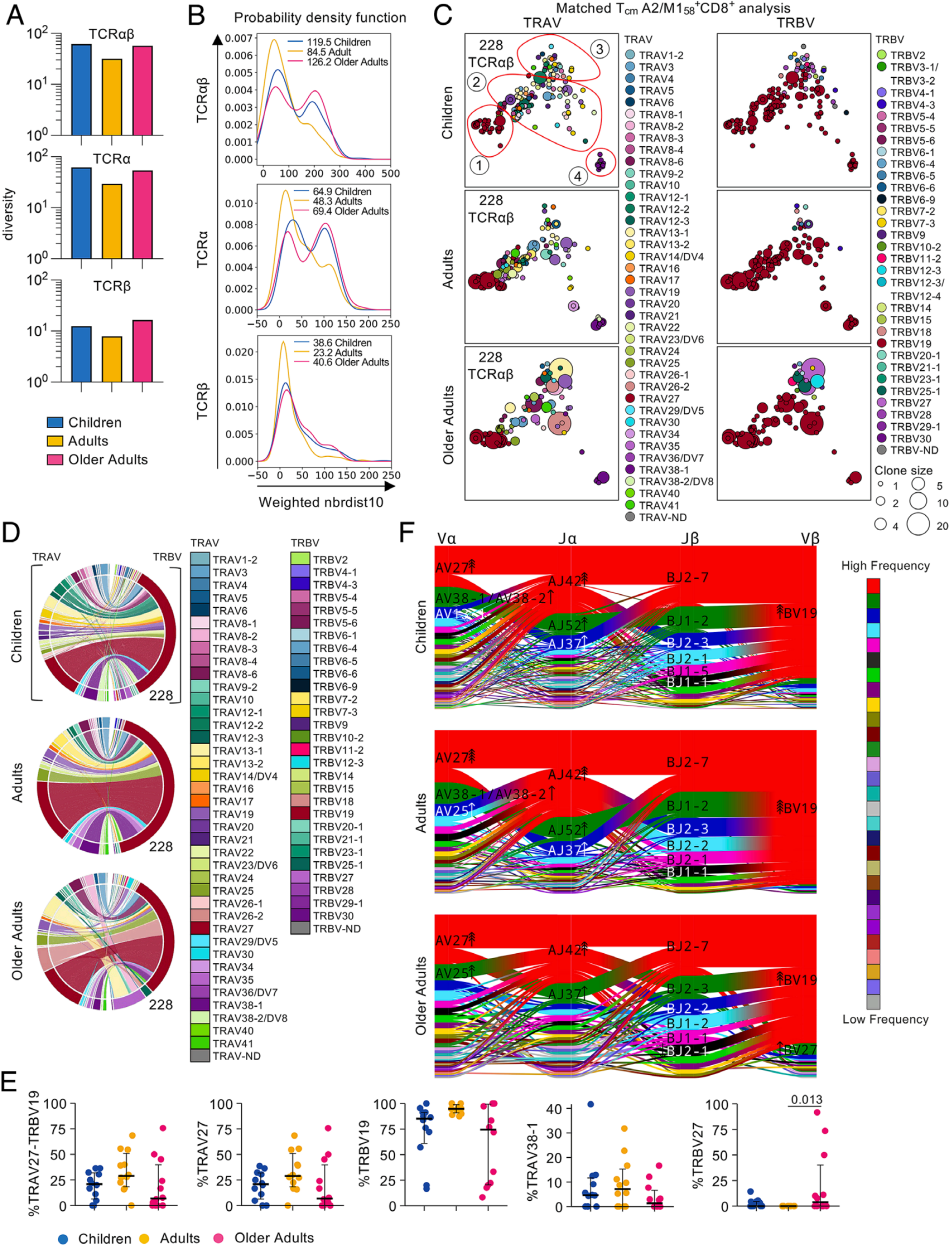
TCRαβ repertoire of T_cm_ A2/M1_58_^+^CD8^+^ T cells in children, adults, and older adults. (*A*) TCR diversity of matched paired TCRαβ-chains, TCRα, and TCRβ measured by TCRdiv. (*B*) Neighbor distance distribution smoothed density profiles of for each age group. More clustered A2/M1_58_^+^CD8^+^ T_cm_ TCRαβ, TCRα, or TCRβ repertoire are indicated by lower distribution peaks, average distance values are depicted within the plot for each age group. PDF, probability density function. (*C*) 2D-kernel principal component analysis (PCA) projection of the paired A2/M1_58_^+^CD8^+^ T_cm_ TCR landscape split by age group. Clone size indicated by symbol size; TRAV and TRBV gene usage indicated by color. Numbers of paired TCRαβ sequences in each plot is displayed on the *Top Left*. Red circles were manually drawn to indicate regions of interest. (*D*) Circos plots of TRAV and TRBV clonotype pairing per age group. Outer arch segment colored by TRAV and TRBV usage. TRAV–TRBV gene pairing indicated by connecting lines which are colored based on their TRAV usage and segmented based on their CRD3α and CDR3β sequence. The thickness is proportional to TCR clone number with the respective pair. The number at the right bottom of the circos plot indicated the number of sequences considered. (*E*) Proportion of sequences expressing TRAV27-TRRBV19, TRAV27, TRBV19, TRAV38-1, or TRBV27. Bars represent median and interquartile range. A two-sided Kruskal–Wallis with Dunn’s test for multiple comparisons was used to determine statistical significance. (*F*) Gene segment landscapes, V- and J-segments usage indicated by vertical stacks, colored by frequency within the TCR repertoire. Curved paths indicate gene–gene pairing and the thickness is proportional to the number of paired TCR clone. Arrows indicate enrichment of gene segments relative to background frequencies; each arrowhead indicates twofold enrichment.

**Fig. 3.**
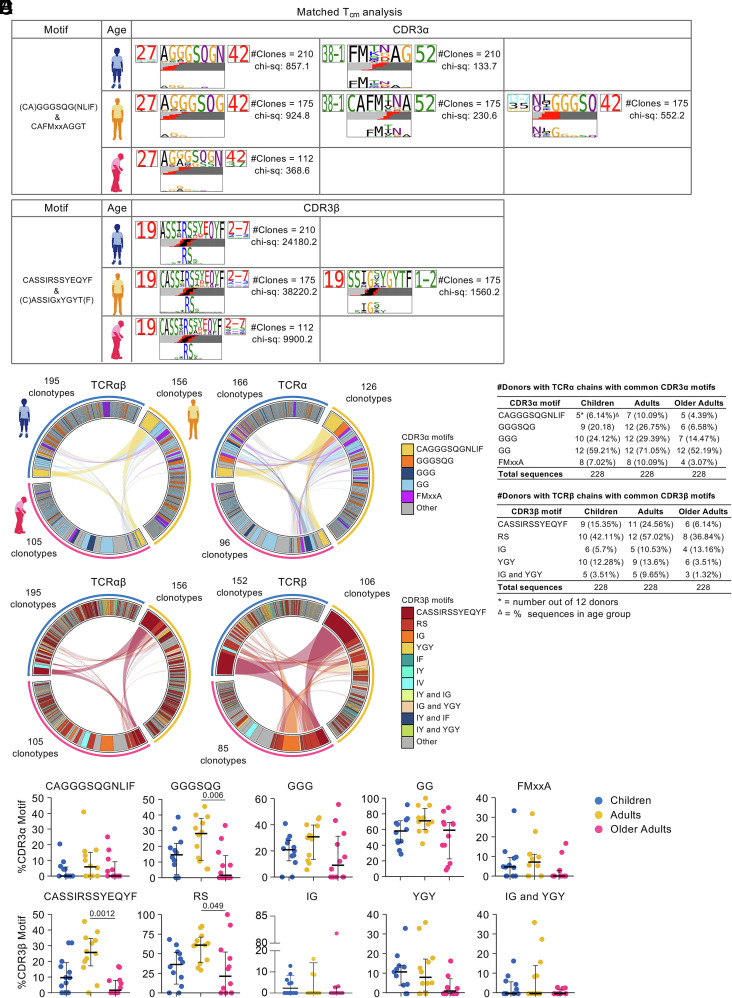
CDR3αβ motifs of T_cm_ A2/M1_58_^+^CD8^+^ T_cm_ across age groups. (*A*) Top-scoring logo representations of matched paired A2/M1_58_^+^CD8^+^ T_cm_ CDR3α and CDR3β sequence motifs across age groups. Each logo shows the V (*Left* side) and J (*Right* side) gene frequencies with CDR3 amino acid sequences in the middle with the full height (*Top*). To highlight motif positions under selection, CDR3 amino acid sequences are scaled by per-residue reparative entropy to background frequencies derived from TCRs with matching gene-segment composition at the *Bottom*. The inferred rearrangement structure by source region (light gray for V-region, dark gray for J, black for D, and red for N insertions) of grouped receptors is shown in the middle. (*B*) Frequency of common CDR3αβ motifs within paired A2/M1_58_^+^CD8^+^ T_cm_ TCR repertoires across age. Connecting lines represent paired TCRαβ (*Left*), TCRα (*Top Right*), or TCRβ-chains (*Bottom Right*) shared between age groups. Plots are colored by CDR3α motif (*Top* row) or CDR3β (*Bottom* row). (*C*) Proportion of common CDR3α motifs or (*D*) CDR3β motifs across children, adults, older adults. A two-sided Kruskal–Wallis with Dunn’s test was used to establish statistical significance for multiple comparisons. (*E*) Number of donors across age group expressing common CDR3αβ motifs. The percentage of sequences expressing these motifs in each age group is in parentheses.

We dissected 814 paired TCRαβ clonotypes within T_cm_ A2/M1_58_^+^CD8^+^ T cells from HLA-A*02:01-expressing newborns (n = 6), children (n = 12), adults (n = 12), and older adults (n = 12) (*SI Appendix*, Table S2). To avoid bias caused by uneven sequence numbers (47), we randomly down-sampled adult and older adult paired TCRαβ sequences to 228 (from 315 and 262, respectively) to match the number of paired TCRαβ sequences in children. We refer to this as “matched T_cm_ analysis” (Figs. 2 and 3 and *SI Appendix*, Figs. S3 and S4 and Dataset S1). We analyzed the full non-downsampled dataset containing paired TCRαβ sequences, single TCRα, and single TCRβ sequences revealing similar findings, referred to as “total T_cm_ analysis” (*SI Appendix*, Figs. S5–S7 and Dataset S2). We excluded newborns from our analysis of the T_cm_ A2/M1_58_^+^CD8^+^ TCRαβ repertoires due to low numbers of paired TCRαβ sequences.

We assessed TCR diversity within T_cm_ A2/M1_58_^+^CD8^+^ repertoires using TCR diversity scores (TCRdiv) calculated by TCRdist (48). TCRdiv is an extension of Simpson’s diversity index (SDI), which considers the similarity of TCRs within each age group. TCRdiv showed that TCRαβ diversity decreased from children (61.6) to adults (31.9) and increased in older adults (57.3) (Fig. 2*A*). This was evident not only for TCRαβ paired clonotypes but also when single TCRα and TCRβ chains were analyzed in the matched T_cm_ analysis (Fig. 2*A*) and in the total T_cm_ analysis (*SI Appendix*, Fig. S5*A*).

To measure TCR repertoire density within each age group and to quantify the contribution of clustered and diverged TCRs, we calculated neighbor distance distributions. More similar clustering of clonotypes is depicted by lower average values of the distance distribution peak. A bimodal distribution was observed for paired TCRαβ-chains and single TCRα-chains in children and older adults (Fig. 2*B* and *SI Appendix*, Fig. S5*B*), while adult TCR repertoire was skewed to the left, indicating an overall lower distribution of the paired TCRαβ-chains and single TCRα-chains (average children αβ:119.5, α:64.9; adults αβ:84.5; α:48.3; older adults αβ:126.2, α:69.4). The TCRβ-chain of all age groups exhibited a single low distribution peak (average children β:38.6; adults β:23.2; older adults β:40.6) (Fig. 2*B* and *SI Appendix*, Fig. S5*B*). These findings were consistent with TCR diversity scores (Fig. 2*A* and *SI Appendix*, Fig. S5*A*), demonstrating greater TCR diversity within T_cm_ A2/M1_58_^+^CD8^+^ T cell pools in children and older adults.

### T_cm_ A2/M1_58_^+^CD8^+^ TCRαβ Repertoire Retains Public-Associated TCRαβ Genes Across Age Groups, with Increased TCRαβ Heterogeneity Among Older Adults.

The common TCRαβ signature of A2/M1_58_^+^CD8^+^ repertoire is the prominent public clonotype detected across HLA-A*02:01-expressing individuals, characterized by TRBV19/complementarity-determining region (CDR)3β-SIRSSYEQ paired with TRAV27/CDR3α-GGSQGNL. We identified other public-associated variable gene usage and CDR3αβ motifs (16, 31, 41). To determine TRAV and TRBV usage within the T_cm_ A2/M1_58_^+^CD8^+^ TCR repertoire, we generated gene segment and gene–gene pairing landscapes for T_cm_ A2/M1_58_^+^CD8^+^ TCRαβ repertoire, across age groups and individual donors (Fig. 2*C*, *SI Appendix*, Figs. S3 and S5). Two-dimensional (2D)-kernel principal component analysis (kPCA) projections of the matched T_cm_ A2/M1_58_-specific TRAV and TRBV gene segments from all age groups revealed four distinct clusters (Fig. 2*C* and *SI Appendix*, Fig. S3*A*). Public-associated TRAV27–TRBV19 expressing TCR clonotypes, including the full public clonotype (16, 31), dominated the cluster on the left (cluster 1), closely located to the TRBV19 dominated cluster in the middle (cluster 2), but paired to diverse TRAV gene segments. TRBV27-expressing TCR clonotypes were prominently featured in the top cluster (cluster 3) and a smaller more distant cluster at the right bottom (cluster 4) consisted mostly of TRAV38-1–TRBV19 expressing clonotypes (Fig. 2*C* and *SI Appendix*, Fig. S3*A*). The four distinct clusters were identified across children, adults, and older adults (Fig. 2*C* and *SI Appendix*, Fig. S5*C*).

Individual differences in each cluster could be observed across age groups (*SI Appendix*, Fig. S3*B*). The public-associated cluster 1 dominated across HLA-A*02:01-positive participants in all age groups, although it was detected in a lower number of older adults [10/12 of children (83%), 11/12 of adults (92%), and 8/12 of older adults (67%)]. Conversely, the TRAV38-1 dominated cluster 4 was more prominent in children and adults (both 8/12, 67%) compared to older adults (4/12, 33%). Although cluster 3 could be detected across the majority of participants (27/36, 75%) across age groups, large clonal expansions in cluster 3 were characteristic for older adults (10/12 children/0 expanded, 8/12 adults/1 expanded, 9/12 older adults/6 expanded). Most donors had TRBV19-expressing clonotypes from cluster 2 (*SI Appendix*, Fig. S3*B*). Overall, age-specific T_cm_ A2/M1_58_^+^CD8^+^ TCRαβ repertoires clustered in similar patterns across the human lifespan, although higher heterogeneity was observed for older adults (*SI Appendix*, Fig. S3*C**)*.

TCR circos analyses revealed that T_cm_ A2/M1_58_-specific TCRs had a high degree of similarity in TRAV and TRBV gene segment usage between age groups (Fig. 2*D* and *SI Appendix*, Figs. S4–S6). TRAV gene segment usage was least diverse among adults, although a strong prevalence for the public-associated TRAV27 gene segment was observed across all age groups. In contrast, T_cm_ A2/M1_58_-specific TCRs of all age groups were dominated by pubic-associated TRBV19-expressing clonotypes, frequently paired with the TRAV27 gene segment, with no significant differences in their TRAV27, TRBV19, or paired gene expression between age groups in the matched T_cm_ analysis, although TRAV27, TRBV19, and paired clonotypes significantly decreased in the total T_cm_ analysis (Fig. 2*E* and *SI Appendix*, Figs. S4–S6 and Datasets S1 and S2). Matching our kPCA, TRAV38-1 was most prominent in children and adults, albeit not significant (Fig. 2 *D* and *E* and *SI Appendix*, Figs. S4, S5 *D* and *E* and S6 and Datasets S1 and S2). However, older adults displayed prominent TRBV27 usage compared to adults, mainly driven by 3 older adults, with large clonal expansions expressing TRBV27 (*SI Appendix*, Figs. S4 and S6). Conversely, only one adult displayed expanded TRBV27-expressing clonotype (in total T_cm_ analysis, *SI Appendix*, Fig. S5 and Dataset S2). TRAV13-1 and TRAV26-2 were prominently featured in older adults T_cm_ A2/M1_58_^+^CD8^+^ TCRαβ repertoire. TRAV13-1 expression stemmed from large clonal expansions in OA1 (paired to TRBV27) and TRAV26-2 expression was driven by large clonal expansions in OA12 and OA19 (paired with TRBV19 and TRBV25-1, respectively) (Fig. 2*D* and *SI Appendix*, Figs. S4 and S5*D* and Datasets S2 and S3).

We dissected correlations between V and J gene segment usage both within chains (Vα-Jα, Vβ-Jβ) and between chains (Vα-Vβ, Jα-Jβ) and quantified gene preferences by comparing gene frequencies within the T_cm_ A2/M1_58_^+^CD8^+^TCRαβ repertoire with a publicly available non-epitope-selected TCR repertoire (48) (Fig. 2*F* and *SI Appendix*, Fig. S5*F*). Public-associated genes TRAV27, TRAJ42, TRBV19, and TRBJ2-7 were commonly paired together, and TRAV27, TRAJ42, and TRBV19 were the top enriched genes across age groups, with 6-fold enrichment in children and older adults and 8-fold in adults (Fig. 2*F* and *SI Appendix*, Fig. S5*F*). TRAJ37 also frequently paired with the TRAV27, TRBJ2-7, and TRBV19 gene segments and with 2-fold enrichment across age groups. Conversely, TRAV38-1/38-2 (2-fold enrichment) with TRAJ52 (fourfold enrichment) were uniquely enriched in children and adults and paired with TRBJ1-2 and TRBV19, whereas TRAV25 was enriched 2-fold in adults and 4-fold in older adults. TRBV27 was uniquely enriched 2-fold in older adults with no clear pairing preference for any TRAV, TRAJ, or TRBV segment (Fig. 2*F* and *SI Appendix*, Fig. S5*F*).

Overall, we show that T_cm_ A2/M1_58_^+^CD8^+^ TCRαβ clonotypes from 4 distinct clusters are maintained across the human lifespan. Although public-associated TCRαβ genes remain prominent, increased heterogeneity observed in older adults is underpinned by large clonotype expansions expressing non-public-associated TCRαβ genes.

### Public-Associated CDR3αβ Motifs Maintained within the T_cm_ A2/M1_58_^+^CD8^+^ T Cell Compartment Despite Shifts in Their Frequencies.

As the hypervariable CDR3α and CDR3β determine fine pHLA-I specificity, we dissected CDR3 regions within the T_cm_ A2/M1_58_^+^CD8^+^ TCRαβ repertoire across the human lifespan. TCRdist (48) identified conserved amino acid residues essential for TCR recognition of the A2/M1_58_ epitope by T_cm_ CD8^+^ cells within each age group. The public CDR3α-associated motif, TRAV27-TRAJ42, glycine-rich, CDR3α-(CA)GGGSQG(NLI) motif and the public-associated TRBV19-TRBJ2-7 CDR3β-”RS” motif dominated the T_cm_ A2/M1_58_^+^CD8^+^ CDR3αβ motifs across all age groups (Fig. 3*A* and *SI Appendix*, Fig. S7*A*). These motifs play key roles for the peg-notch mode of recognition of the A2/M1_58_ epitope (48) and have high TCR avidity, lower activation threshold, compared to less public/more private clonotypes (16). Although CDR3α-”CAGGGSQGNLIF” and CDR3β-CASSIRSSYEQYF motifs became less frequent in older adults, this was only significant for CDR3β-”CASSIRSSYEQYF” expressing clonotypes (Fig. 3 *B*–*E* and *SI Appendix*, Fig. S7 *B–E*). Clonotypes expressing shorter counterparts, CDR3α-”GGGSQG” and CDR3β-RS also decreased, with a similar trend for CDR3α-”GGG” and CDR3α-”GG” which were only significant in total T_cm_ analysis (Fig. 3 *B*–*E* and *SI Appendix*, Fig. S7 *B–E*). We identified the public-associated TRBV19-TRBJ1-2-CDR3β-”IGxYGY” motif in adults (Fig. 3*A*) and the children’s full T_cm_ TCR repertoire (*SI Appendix*, Fig. S7*A*). Clonotypes expressing the public-associated CDR3β-”IG”, “YGY” or “IG and YGY” motifs became less prominent in older adults (Fig. 3 *B* and *D* and *SI Appendix*, Fig. S7 *B* and *D*). CDR3β-”(I)F” motif was only identified in children when analyzing the full T_cm_ TCR repertoire (*SI Appendix*, Fig. S7*A*), but was detected across all age groups in total A2/M1_58_^+^CD8^+^ TCR repertoire (16). We did not detect the TRBV19-associated CDR3β-”IV” and TRBV19-associated CDR3β-”IY” (Fig. 3*A* and *SI Appendix*, Fig. S7*A*), previously identified in total A2/M1_58_^+^CD8^+^ T cell populations (16), suggesting that these CDR3β motifs may be prominent in another memory subset. Similarly, TRAV13-2/TRAV35-TRAJ42-associated CDR3α-GGGSQ motif was detected in adults but only in our down-sampled “matched” T_cm_ A2/M1_58_^+^CD8^+^ T cell analysis (Fig. 3*A*). A similar TRAV12-1/TRAV8-1-TRAJ42-associated “NxGGGSQ” motif was found in children in total T_cm_ A2/M1_58_^+^CD8^+^ analyses (*SI Appendix*, Fig. S7*A*).

We identified a previously described public motif, TRAV38-1-TRAJ52-associated CDR3α-”FMxxA” (42), enriched in T_cm_ A2/M1_58_^+^CD8^+^ T cells of children and adults, but not in older adults (Fig. 3*A* and *SI Appendix*, Fig. S7*A*). As this motif was not seen in our previous total A2/M1_58_^+^CD8^+^ T cell analysis, it may not be prevalent among other memory phenotypes (16) and more specific to T_cm_ pools.

Overall, we found that T_cm_ pools of influenza-specific CD8^+^ T cells were enriched for the public-associated CDR3αβ motifs and preserved across the human lifespan.

### Memory A2/M1_58_^+^CD8^+^ T Cell Subsets Are Clonally Related.

**Fig. 4.**
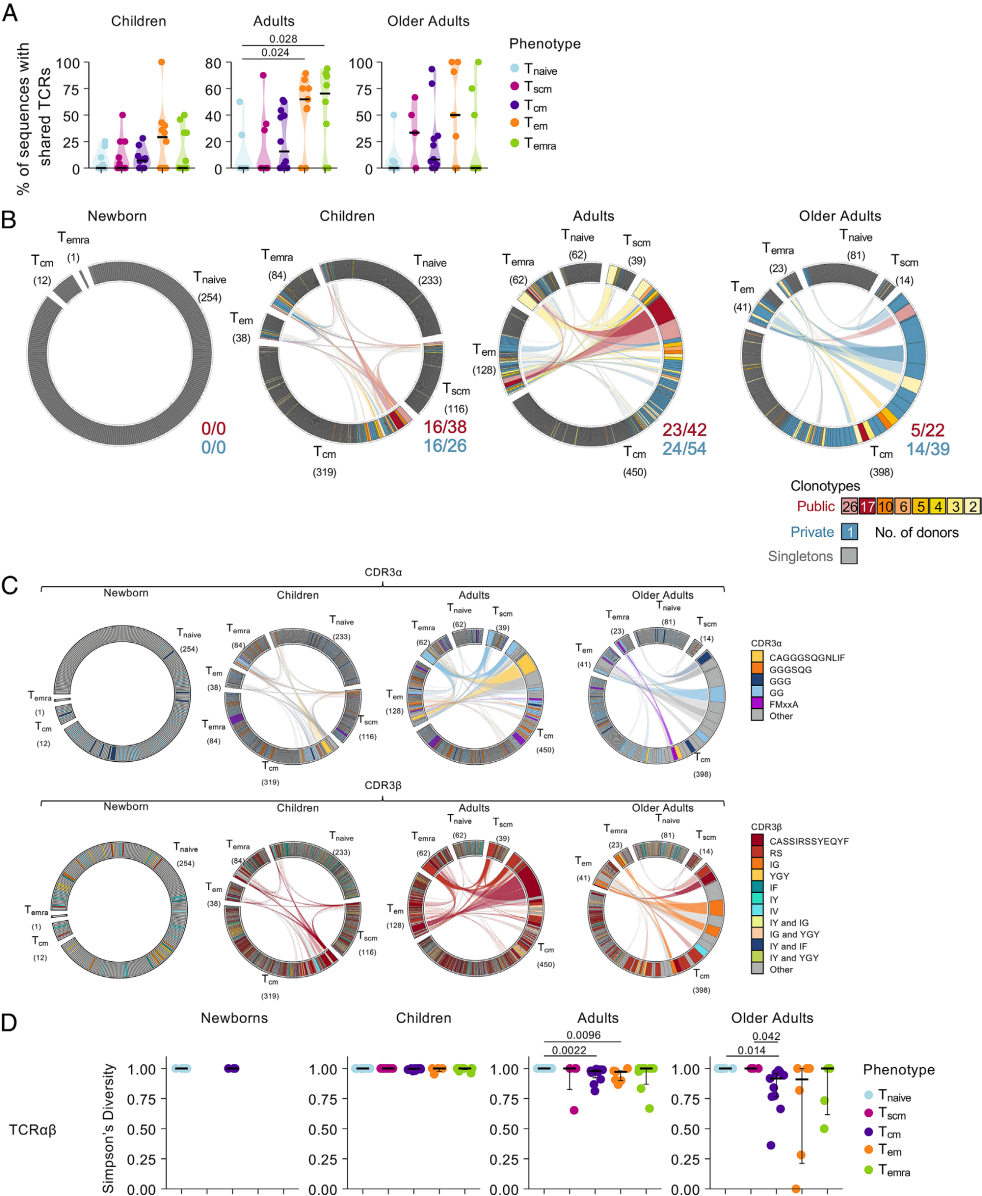
Sharing of public and private clonotypes between memory subsets across age groups. (*A*) Proportion of TCRs shared with another phenotype subset across groups. Statistical significance was established using a two-sided Kruskal–Wallis with Dunn’s test for multiple comparisons. (*B*) Frequency of high-prevalent (>1 similar TCR within a single individual) public (shared) and private (not shared) clonotypes across different phenotypes per age groups. Connecting lines represent paired TCRαβ-chains shared between phenotype subset. Public clonotypes are colored in red, orange, and yellow, private clonotypes in blue. The high-prevalent public TCR (TRAV27, TRAJ42, CDR3α-GAGGGSQGNLIF, TRBV19, TRBV2–7, and CDR3β CASSIRSSYEQYF) is depicted in dark red, whereas clonotypes expressing the full public TCRβ chain (TRBV19, TRBV2–7, and CDR3β-CASSIRSSYEQYF) with an unidentified TCRα-chain are depicted in light red. Each plot represents an age group. The fraction of public (red) or private (blue) TCRαβ clonotypes shared across phenotypes out of the total number of public or private TCRαβ clonotypes are shown on the *Bottom Right*. (*C*) Frequency of common CDR3αβ motifs across different phenotypes per age group. Connecting lines represent paired TCRαβ-chains shared between each memory subset. Plots are colored by CDR3α (*Top*) or CDR3β (*Bottom*) motifs. Each plot represents an age group. (*D*) SDI of paired TCRαβ-chains comparing memory subsets within each age group. Statistical significance was established using a two-sided Kruskal–Wallis with Dunn’s test for multiple comparisons.

The clonal relationship between antigen-specific CD8^+^ T_cm_ pools with other memory subsets across the human lifespan has not been resolved. We thus analyzed the sharing of all TCRαβ clonotypes between memory subsets within A2/M1_58_^+^CD8^+^ T cells across age groups. In children, clonotype sharing was relatively low, with the highest level of sharing with other phenotypes observed within T_cm_ (7.08%) and T_em_ (29.17%) (Fig. 4*A*). In adults, high levels of TCR clonotype sharing was observed among more differentiated memory subsets (T_cm_ 12.50%, T_em_ 51.91%, T_emra_ 56.25%). T_em_ and T_emra_ cells showed significantly more sharing than T_naïve_ cells. For older adults, TCR clonotype sharing was more prominent in the less differentiated T_scm_ subset (33.33%), higher than the T_cm_ subset (8.01%) but lower than the T_em_ subset (50%) (Fig. 4*A*). Within each memory subset, we did not observe any differences in TCR sharing between age groups (*SI Appendix*, Fig. S8*B*).

To define how public and private TCRαβ clonotypes are distributed across different memory A2/M1_58_^+^CD8^+^ T cell subsets, we analyzed distribution of high prevalent public (>1 donor and >1 sequence in at least one donor), and high prevalent private clonotypes (not shared among donors, >1 sequence in a single individual). Increased sharing of expanded public clonotypes across phenotype subsets was observed from children (6/12 donors; 16 out of 38 expanded public TCRαβ clonotypes shared across phenotypes) to adults (7/12 donors; 23/42) and decreased in older adults (3/12 donors; 5/22). Surprisingly, expanded private clonotype sharing across phenotypes was highest in children (children: 16/26, adults: 24/54, and older adults: 14/39) (Fig. 4*B*). However, the number of donors with shared private TCRs was highest in older adults (9/12 donors) compared to children (8/12) and adults (7/12) (*SI Appendix*, Fig. S8*A* and Dataset S3). T_cm_ and T_em_ sharing of public clonotypes peaked in adults (5/12) compared to children and older adults (both 1/12). Whereas T_cm_ and T_em_ sharing of private clonotypes was lowest in children (4/12 children, 6/12 adults, 5/12 older adults) (*SI Appendix*, Fig. S8*A* and Dataset S3). Sharing between other memory phenotypes (T_scm_ and T_emra_) was also observed in individual participants, although these populations were less prominent.

These data suggest that memory T cell subsets are not clonally distinct and that patterns of TCR sharing within influenza-specific A2/M1_58_^+^CD8^+^ TCR αβ repertoires change across the human lifespan.

### A2/M1_58_^+^CD8^+^ T_cm_ Pools Are Clonally Stable Across the Human Lifespan.

As public A2/M1_58_^+^CD8^+^ TCRαβ clonotypes were shared between memory subsets, we investigated whether other A2/M1_58_^+^CD8^+^ T cell clonotypes expressing public-associated CDR3αβ motifs were also shared among memory subsets (Fig. 4*C* and *SI Appendix*, Figs. S9–S11).

The full-public CDR3α-”CAGGGSQGNLIF” sequence peaked in the T_cm_ subsets of children (5/12 children), adults (9/12 adults), and older adults (5/12 older adults), with limited detection in other subsets in children (T_naive_ 1/12; T_scm_ 1/12) and adults (T_em_ 3/12; T_emra_ 2/12), but no subset sharing within older adults (*SI Appendix*, Figs. S9 and S10 and Dataset S3). Shorter variants of the public-associated CDR3α motif (GGGSQG and GGG) were also commonly shared among memory subsets in children and adults, but not in older adults. Conversely, the shortest version of the public-associated CDR3α motif-”GG” was detected across memory subsets in children, adults, and older adults.

The newly identified young T_cm_ A2/M1_58_^+^CD8^+^ T cell–associated CDR3α-”FMxxA” motif was detected in children’s T_scm_ (5/12), T_cm_ (8/12), and T_emra_ (1/12) subsets but CDR3α-FMxxA were not shared between subsets of individual children (*SI Appendix*, Figs. S9 and S10 and Datasets S3 and S4). The CDR3α-FMxxA motif was detected in all phenotype subsets in adults (T_naive_ 2/12, T_scm_ 1/12, T_cm_ 9/12, T_em_ 5/12, T_emra_ 2/12), and with some clonotypes containing this motif being shared between T_cm_ and T_em_ populations in 2/12 adults. In older adults, the CDR3α-FMxxA motif was detected in T_cm_ (4/12), T_em_ (2/12), and T_emra_ (1/12), with sharing between T_cm_, T_em_, and T_emra_ in 1/12 older adults (*SI Appendix*, Figs. S9 and S10 and Datasets S3 and S4).

The full public CDR3β-”CASSIRSSYEQF” sequence was shared between all phenotype subsets in children (T_naive_ 4/12, T_scm_ 4/12, T_cm_ 9/12, T_em_ 5/12, T_emra_ 5/12) (Fig. 4*C* and *SI Appendix*, Figs. S9 and S11 and Datasets S3 and S4). In adults, the full public CDR3β sequence was restricted to and shared among the more differentiated subsets (T_cm_ 12/12, T_em_ 7/12, T_emra_ 6/12), while in older adults it was mainly restricted to T_cm_ pools (8/12) and shared between T_scm_ and T_em_ in one older adult. The public-associated CDR3β-RS motif was detected across all phenotypes in children, adults, and older adults (*SI Appendix*, Figs. S9 and S11 and Datasets S3 and S4). While sharing among all memory subsets increased from children (1/12) to adults (6/12), the sharing in older adults was limited to 3/12 donors. Other public-associated CDR3β motifs including the IG, YGY, and IG and YGY motifs were detected across all phenotypes in children and adults but shared to a limited extent. The IG-motif was prominently featured and shared among the T_cm_, T_em_, and T_emra_ subsets of one older adult. The “YGY” motif could be detected in 4/12 older adults but was shared between T_cm_ and T_emra_ cells in one older adult. The combined “IG and YGY” motif was detected at low frequencies in the T_cm_ population of 4/12 older adults but not in other phenotypes (*SI Appendix*, Figs. S9 and S11 and Dataset S3 and S4).

Overall, our data suggest prominent public-associated TCR clonotypes are maintained among T_cm_ pools across different ages, which may replenish the T_em_ and T_emra_ compartments.

### TCR Diversity Decreases in More Differentiated Memory Phenotypes with Increasing Age.

To investigate the hierarchy of memory CD8^+^ T cell subsets, we evaluated TCR diversity by comparing the SDI in each subset within each age group (Fig. 4*D*). In adult and older adult A2/M1_58_^+^CD8^+^ T cells, there was a trend for lower TCRαβ diversity in more differentiated subsets (T_em_, T_emra_), even more pronounced in individual TCRα and TCRβ chains (Fig. 4*D* and *SI Appendix*, Fig. S12*A*). This corresponded with the observed increase in shared TCRαβ clonotypes from T_naive_ to more differentiated memory subsets from children to adults, with significantly higher shared frequencies in adult T_em_ and T_emra_ populations (Fig. 4*A*).

Overall, patterns in TCRαβ diversity and TCR sharing support the decreasing potential model of CD8^+^ T cell memory lineage relationship, which seems to be more prominent with age, likely reflecting age-related changes in CD8^+^ T cell differentiation.

### Age-Related Decrease in TCRαβ Diversity within the A2/M1_58_^+^CD8^+^ T_cm_ Population Associated with Private Clonotype Expansions.

Given uneven distribution of private clonotypes among memory subsets, we hypothesized that increased expanded private clonotypes may drive decreased TCR diversity across the human lifespan. There was a negative correlation between TCRαβ diversity and age in A2/M1_58_^+^CD8^+^ T_cm_ cells, which was also observed in the single TCRα- and β-chains (*SI Appendix*, Fig. S12*B*). A trend for a similar negative correlation was observed for the T_em_ and T_emra_ population; significant for the single T_em_ TCRα-chain. Heterogeneity in TCRαβ diversity increased from children to older adults, with some older adults maintaining a higher diversity. Heterogeneity in adult T_cm_ A2/M1_58_^+^CD8^+^ cells was determined by the prevalence of public clonotypes (*SI Appendix*, Fig. S13*A*), in contrast to children and older adults where heterogeneity in T_cm_ A2/M1_58_^+^CD8^+^_,_ T_em_ A2/M1_58_^+^CD8^+^_,_ and T_emra_ A2/M1_58_^+^CD8^+^ T cells was linked to increased private clonotypes (*SI Appendix*, Fig. S13*B*). Thus, private clonotypes that become more prevalent with age are mainly detected within T_cm_ and T_em_ and reduce the TCR diversity of T_cm_ pools.

### A2/M1_58_^+^CD8^+^ T Cell Polyfunctionality Is Subset Dependent.

To assess functionality of memory A2/M1_58_^+^CD8^+^ T cells with age, we mined our published data on 9 d in vitro expanded A2/M1_58_^+^CD8^+^ T cells, restimulated with M1_58-66_ peptide and stained intracellularly for IFN-γ, TNF, Perforin, and Granzyme B (16). On day 0, children, adults, and older adults A2/M1_58_^+^CD8^+^ T cells predominantly displayed the T_cm_ subset (*SI Appendix*, Fig. S14*A*). By day 9, A2/M1_58_^+^CD8^+^ T cells mostly differentiated into T_em_. On day 9, a significantly higher percentage (28.6%) of T_cm_ in children expressed IFN-γ, Perforin, and Granzyme B compared to adults (8.7%) and older adults (0%). However, the most polyfunctional T_cm_ population, expressing IFN-γ, TNF, Perforin, and Granzyme B, was observed in adults (22.8%) compared to children (8.1%) and older adults (8.1%), albeit not significant (*SI Appendix*, Fig. S14*B*). Conversely, the T_em_ and T_emra_ subsets were most polyfunctional in children, with 32.9% T_em_ and 31.1% T_emra_ expressing IFN-γ, TNF, Perforin, and Granzyme B, followed by adults (26.2% T_em_ and 8.3% T_emra_) and then older adults (12.1% T_em_ and 0% T_emra_), a pattern that closely resembled what we observed in the total A2/M1_58_^+^CD8^+^ T cell population (16). Although we did not observe differences in protein expression directly ex vivo (*SI Appendix*, Fig. S2*B*), these data confirm the higher effector potential of younger T_cm_ cells observed in our scRNAseq data (Fig. 1 *H* and *I*).

## Discussion

We analyzed the prevalent memory T cell subsets in human blood, directed at the predominant A2/M1_58_ epitope, in newborns, children, adults, and older adults ex vivo. We demonstrated that T_cm_ CD8^+^ pools dominate virus-specific memory A2/M1_58_^+^CD8^+^ T cell responses from the early childhood until old age, with prominent overlap in TCR repertoires and gene expression profiles. Our study highlights the importance of T_cm_ pools across the human lifespan and advocates for boosting persistent TCRαβ clonotypes within this key blood subset.

T_cm_ A2/M1_58_^+^CD8^+^ displayed minimal transcriptomic changes across the human lifespan, although self-renewal genes defined T_cm_ pools in children, while older T_cm_ A2/M1_58_^+^CD8^+^ T cells displayed detoxication and stress profiles. Children’s T_cm_ A2/M1_58_^+^CD8^+^ cells were less differentiated and displayed a signature indicating potent T cell activation and survival potential. Older adult T_cm_ had reduced effector expression profile which was not reflected at the protein level directly ex vivo. However, when looking at effector functions upon in vitro stimulation, effector profiles were more prominent in children and decreased with increasing age. Older adult T_cm_ displayed increased expression of genes involved in TCR activation, detoxification, and stress–response and were enriched with a stress signature. Stress–response and cellular detoxification may aid in dealing with reactive oxygen species which can induce terminal differentiation and senescence (49, 50), potentially contributing to the relatively low prevalence of A2/M1_58_^+^ CD8^+^ T_emra_ populations in older adults (16).

TCRαβ diversity within T_cm_ A2/M1_58_^+^CD8^+^ T cells was highest in children and older adults. Key public-associated TCRαβ clonotypes largely persisted across the human lifespan in T_cm_ A2/M1_58_^+^CD8^+^ T cells. Older adults displayed increased TCRαβ heterogeneity, underpinned by expansions of private TCRαβ clonotypes. Given that the A2/M1_58_ epitope is highly conserved (51), it is unlikely that reduced TCRαβ diversity would be detrimental in the context of this epitope. However, lower TCRαβ diversity in T_cm_ A2/M1_58_^+^CD8^+^ T cells was associated with higher degrees of private clonotype expansions, which have suboptimal avidity to the A2/M1_58_ epitope (16).

Memory subsets were clonally related with more differentiated subsets consisting of more shared TCRs, conversely to the distinct lineage model. Both public and private A2/M1_58_^+^CD8^+^ TCRαβ clonotypes were shared between memory subsets from childhood through to old age. Private clonotypes were particularly expanded within the T_cm_ compartment in older adults and were linked to lower TCR diversity. More differentiated memory subsets displayed reduced TCR diversity, supportive of the decreasing potential model.

A2/M1_58_^+^CD8^+^ T_cm_ TCRαβ repertoires overlapped between age groups although they were more heterogenous among older adults. Notably, the public associated TCRαβ clonotypes (16, 41) were observed in the A2/M1_58_^+^CD8^+^ T_cm_ TCRαβ repertoire across age, highlighting importance of T_cm_ in maintaining functional clonotypes in older adults. Both public-associated and private TCRs were shared between phenotypes. The importance of T_cm_ was further exemplified by the observation that sharing of clonotypes was the highest between T_cm_ and other memory subsets, particularly T_em_ and T_emra_ pools.

The decreasing potential model predicts that TCR signaling strength and duration drives CD8^+^ T cell differentiation (52) such that “optimal high avidity TCRs” dominate the effector phase. A consequence of this would be a decline in TCR diversity in more differentiated memory subsets (T_naive_>T_scm_>T_cm_>T_em_>T_emra_). This pattern was observed in adults and older adults, supportive of this model and experiments in mice, showing higher TCR diversity in T_cm_ than in T_em_ pools (21). However, it is unclear whether T_emra_ cells represent the end differentiation stage, as these CD8^+^ T cells had similar TCR diversity as the T_em_ pools. Additionally, the decreasing potential model predicts that the proportion of TCRs that are shared should be the highest in more differentiated memory subsets (T_naive_<T_scm_<T_cm_<T_em_<T_emra_). This was observed for adults; however, children did not fit into the same patterns of TCR diversity and sharing as adults. There are several possible explanations for this discrepancy. First, repeated infections with IAV lead to a more pronounced manifestation of the decreasing potential model of memory generation. Second, children who have a highly functional thymus may benefit from novel TCR clonotypes entering their TCR repertoire as naïve cells. Since the addition of novel TCR clonotypes decreases with age due to thymic involution, it may result in a more pronounced decreasing potential phenotype over time. Our results contrast a study that found that CMV-specific CD8^+^ T cells had lower TCR diversity in T_scm_ versus T_cm_ and T_em_ (53), likely reflecting differences in T cell differentiation in acute infections like influenza versus chronic infections like CMV (54). Given T cells may utilize a mix of models to differentiate (26), some have suggested that asymmetric division may act to guarantee memory formation in situations of strong TCR stimulation that may drive T cell differentiation (55).

Our study shows that T_cm_ are the key important reservoir of persistent TCRs across the human lifespan and provides insights into the TCR clonal relationship between memory subsets.

Long-lived T_cm_ subsets are the key population to boost or induce persistent CD8^+^ T cell populations. It is important to note that prior influenza vaccination is not a factor for our study, as Australia only uses inactivated influenza vaccines not inducing CD8^+^ T cell responses (56). Further understanding of how CD8^+^ T cells differentiate into T_cm_ would allow for the generation of vaccines that target long-lived and highly functional T cell populations.

### Limitations of the Study.

Our study focuses on T_cm_ CD8^+^ T cells specific to a dominant IAV epitope (A2/M1_58_). Further work is needed to understand T_cm_ CD8^+^ T cells toward other viruses and epitopes. The timing of influenza virus infection is unknown for our cohort. Previous seroprevalence studies have shown that 50% of individuals will have had an IAV infection by the age of 2, and close to 100% by the age of 7 (57). We have not performed a power calculation, as our sample size was determined by sample availability from HLA-A*02:01-expressing healthy individuals across the human lifespan.

## Materials and Methods

### Study Participants and Ethics.

Our study included healthy HLA-A*02:01 participants (*SI Appendix*, Table S1). Adults and older adults were recruited via University of Melbourne (UoM), Deepdene Medical Clinic (DMC; Australia), and from our previous study (16). Buffy packs were obtained from Australian Red Cross Lifeblood (Melbourne, Australia); children via Launceston General Hospital (Tasmania, Australia), St Jude Children’s Research Hospital (Memphis, USA). Umbilical cord bloods (Mercy Hospital for Women (Heidelberg, Victoria) were recruited from a previous study (16). All participants provided informed written consent. Experiments conformed to the Declaration of Helsinki Principles and the NHMRC Code of Practice. Ethical approval was provided by the Human Research Ethics Committee of UoM (ethics IDs #24567, #13344, #23852), Australian Red Cross Lifeblood (2015#8), St Jude Children’s Research Hospital (XPD12-089 IIBANK), Mercy Hospital for Women (#R14-25), Tasmanian Health and Medical Human Research Ethics Committee (#H0017479). We used PBMCs from HLA-A*02:01^+^ newly recruited adults (10 participants) and older adults (6 participants), with analyses of the data from our HLA-A*02:01^+^ cohort (16).

## Supplementary Material

Appendix 01 (PDF)

Dataset S01 (XLSX)

Dataset S02 (XLSX)

Dataset S03 (XLSX)

Dataset S04 (XLSX)

Dataset S05 (XLSX)

Dataset S06 (XLSX)

## Data Availability

TCR sequence data (Datasets S1, S2, S3 and S4) has been deposited into Mendeley [https://doi.org/10.17632/vmxby5r95w.1] (58) and VDJdb (https://vdjdb.cdr3.net) (59). scRNASeq data are available from the NCBI Gene Expression Omnibus under the Accession code GSE237817 (16). All other data are included in the manuscript and/or supporting information.
