# Supplementary material for: Central memory T cells with key TCR repertoires and gene expression profiles dominate influenza CD8+ T cell pools across the human lifespan
Source: Proc Natl Acad Sci U S A. 2025 Jul 22;122(30):e2501167122. doi: 10.1073/pnas.2501167122 (PMC12318230; doi:10.1073/pnas.2501167122)
Supplement: Supplementary file 1 — Appendix 01 (PDF) [file pnas.2501167122.sapp.pdf]

Central memory T-cells with key TCR repertoires and gene expression profiles dominate influenza CD8+ T-cell pools across human lifespan

Tejas Menon[a], Hayley A. McQuilten[a], Jerome Samir[b], Thi H.O. Nguyen[a], Ratana Lim[c], Jasveen Kaur[d], Simone Rizzetto[b], Auda Eltahla[2], Paul G. Thomas[e], Martha Lappas[c], Jamie Rossjohn[f,g], Stephanie Gras[f,h], Jane Crowe[i], Katie L. Flanagan[d,j,k], Fabio Luciani[b], Peter C. Doherty[a,1,2], Carolien E. van de Sandt[a,1] and Katherine Kedzierska[a,1,2]

Contributed by Peter C. Doherty

[a]Department of Microbiology and Immunology, University of Melbourne, at the Peter Doherty Institute for Infection and Immunity, Parkville, Australia.
[b]School of Medical Sciences and The Kirby Institute, UNSW Sydney, Sydney, New South Wales, Australia.
[c]Obstetrics, Nutrition and Endocrinology Group, Department of Obstetrics and Gynaecology, University of Melbourne, Melbourne, Victoria, Australia.
[d]School of Health Sciences and School of Medicine, University of Tasmania, Launceston, Tasmania, Australia.
[e]Department of Host-Microbe Interactions, St. Jude Children's Research Hospital, Memphis, TN, USA.
[f]Immunity Program and Department of Biochemistry and Molecular Biology, Biomedicine Discovery Institute, Monash University, Clayton, Victoria, Australia.
[g]Institute of Infection and Immunity, Cardiff University School of Medicine, Cardiff, UK.
[h]Viral and Structural Immunology Laboratory, Department of Biochemistry and Chemistry, La Trobe Institute for Molecular Science, La Trobe University, Bundoora, Victoria, Australia.
[i]Deepdene Surgery, Deepdene, Victoria, Australia.
[j]School of Health and Biomedical Science, RMIT University, Melbourne, Victoria, Australia.
[k]Tasmanian Vaccine Trial Centre, Clifford Craig Foundation, Launceston General Hospital, Launceston, Tasmania, Australia.
[1]These authors contributed equally
[2]Correspondence; pcd@unimelb.edu.au and kkedz@unimelb.edu.au

Supplementary information text

Figure S1. Gating strategy to identify A2/M1$_{58}$$^+$CD8$^+$ T cells and their memory subsets.
Figure S2. scRNASeq analysis and validation.
Figure S3. TCR repertoire of matched T$_{cm}$ A2/M1$_{58}$$^+$CD8$^+$ cells across age groups.
Figure S4. Circos analysis of matched T$_{cm}$ A2/M1$_{58}$$^+$CD8$^+$ cells in individual donors.
Figure S5. TCRαβ repertoire of total A2/M1$_{58}$$^+$CD8$^+$ T$_{cm}$ across age groups.
Figure S6. Circos analysis of total A2/M1$_{58}$$^+$CD8$^+$ T$_{cm}$ in individual donors.
Figure S7. CDR3αβ motifs of total A2/M1$_{58}$$^+$CD8$^+$ T$_{cm}$ across age groups.
Figure S8. Sharing of public and private clonotypes between phenotype subsets across individual participants.
Figure S9. Frequency of common CDR3αβ motifs in each phenotype subset across age groups.
Figure S10. Frequency of common CDR3α motifs across phenotype subsets per individual participant.
Figure S11. Frequency of common CDR3β motifs across phenotype subsets per individual participant.
Figure S12. TCR diversity across age groups.
Figure S13. TCR diversity across age groups in each memory T-cell subset.
Figure S14. Polyfunctionality of T$_{cm}$ and T$_{em}$ A2/M1$_{58}$$^+$CD8$^+$ T-cell.

Table S1 Demographics of healthy HLA-A*02:01-expressing participants in our study
Table S2 Total number of T$_{cm}$ A2/M1$_{58}$$^+$CD8$^+$ TCRs.

Supplementary information text

METHODS

Ex vivo A2/M1$_{58}$ tetramer enrichment

PBMCs were thawed out before tetramer associated magnetic enrichment (TAME) (1-4). Briefly, PBMCs (10–50×10^6) were blocked with FcR block (Miltenyi Biotec) for 15 mins, stained with A2/M1$_{58}$ tetramer at room temperature for 1h, incubated with anti-PE microbeads (Miltenyi Biotec) before enriching the A2/M1$_{58}$ tetramer-positive cells by magnetic separation through LS columns (Miltiney Biotec). For some experiments, anti-CXCR4-BUV395 (1:50, BD Horizon #563924) was added in the last 30 minutes of the tetramer stain. Cells were then stained with human anti-CD71-BV421 (1:50, BD Horizon #562995) or anti- CD69 (1:200, BioLegend #310930), anti-CD3-BV510 (1:200, BioLegend #317332), anti-HLA-DR-BV605 (1:100, BioLegend #307640), anti-CD4-BV650 (1:200, BD Horizon #563875), anti-CD27-BV711 (1:200, BD Horizon #563167), anti-CD38-BV786 (1:100, BD Horizon #563964), anti-CD57-APC (1:400, BD Biosciences #560845), anti-CCR7-AF700 (1:50, BD Pharmingen #561143), anti-CD14-APC-H7 (1:100, BD Pharmingen #560180), anti-CD19-APC-H7 (1:100, BD Pharmingen #560177 or #560252), Live/Dead fixable NIR dead-cell stain (1:800, Invitrogen #L34976), anti-CD45RA-FITC (1:200, BD Pharmingen #555488) or anti-CD45RA-BUV805 (1:100, BD #742020) or anti- TRBV19-FITC (IO test, IM1234), anti-CD8-PerCP-Cy5.5 (1:50, BD Pharmingen #565310) or anti-CD8-BV605 (1:200, BD Horizon #564116), anti-CD95-PE-CF594 (1:100, BD Horizon #562395) and anti-PD-1-PE-Cy7 (1:50, BD Pharmingen #561272) (1). Cells were resuspended in MACS for single-cell index sorting using FACSAriaIII or fixed with 1% PFA. For some experiments, cells were fixed and permeabilised with the FoxP3/transcription factor staining kit (Invitrogen, #00-5523-00) and intracellularly stained with anti-NKG7-PE (1:100, Beckman Coulter IM3293), anti-Perforin-PE-Cy7 (1:10, Biolegend #353316) or anti-Perforin-BV421 (1:50, Biolegend #353307), anti-Granulysin-PE (1:50, Biolegend #348003) and anti-Granzyme A-PE-Cy7 (1:50, Biolegend #507222). Samples were acquired on LSRII Fortessa; then analyzed with FlowJo v.10.8.1. Samples with <10 A2/M1$_{58}^{+}$ CD8^{+} T-cells counted were excluded from phenotypic analysis. Data from 11 newborns, 12 children, 20 adults and 18 older adults were obtained from our previous dataset (1).

Tetramer^{+} T cell frequencies were calculated relative to the total CD8^{+} T cell numbers in an unenriched fraction as described previously (5,6), using the following calculation

$$\frac{\#\ Tetramer^{+}CD8^{+}\ T\ cells\ in\ enriched\ fraction}{(Total\ counted\ lymphocytes\ in\ sample\ \times\ \%CD8^{+}\ T\ cells\ in\ unenriched\ fraction)\ /\ 100}$$

Single-cell RNA sequencing (scRNASeq) analysis

Gene expression datasets from 3 newborns, 3 children, 3 adults and 3 older adults were described in our previous study (1). A2/M1$_{58}^{+}$CD8^{+} T-cells and their gene expression and TCR data were manually grouped into T-cell memory subsets ($T_{naïve}$, T_{scm}, T_{cm}, T_{em} and T_{emra}) based on CD45RA, CD27 and CD95 expression within the single cell index sorting profiles from the previous study (1). Dimensionality reduction was not performed due to limited numbers of T_{cm} (130 in children, 142 in adults and 82 in older adults for a total of 354 T_{cm}) and because we could not distinguish separate T_{cm} clusters in our previous analysis (1). Differential expression output across all the analyses is reported in Dataset S5.

Gene set enrichment analysis (GSEA)

GSEA was performed using the R package fsgea (v.1.30.0) as previously described (1). Briefly, we used tje fgsea(…, maxSize = 500) function across the curated Molecular Signatures Database (MSigDB) Hallmark, C2 curated gene sets consisting of canonical gene sets and our own customised gene signatures (manually curated from published data) for T cell phenotypes (Dataset S6).

Single-cell RT-PCR multiplex paired TCRαβ sequencing

TAME enriched A2/M1$_{58}$$^{+}$CD8^{+} T-cells of 4 new adults and 2 new older adults were single cell index-sorted into 96-well twin.tec PCR plates (Eppendorf) and stored at -80°C. Single-cell paired TCRαβ analysis was performed by multiplex-nested polymerase RT-PCR as described (1). Additional TCR data came from our previous study (1). Sequences were analyzed with FinchTV. V-J regions were identified with IMGT (www.imgt.org/IMGT_vquest). The TCRdist analytical pipeline (7) was used to parse TCR sequences. Clonotypes were defined as single-cell TCRαβ pairs that exhibit the same V, J and CDR3 regions. CDR3 motifs were computed by the TCRdist algorithm and are reflective of CDR3 sequences of variable lengths. Circos plots were generated with the circlize package (8) in Rv.4.4.0 (Comprehensive R Archive Network (CRAN)). The sample_n function in the dplyr package (9) in R was used to downsample T_{cm} sequences.

A2/M1$_{58}$ $^{+}$CD8^{+} T cell proliferation analysis

Data for proliferation/polyfunctionality is from our previous analysis (1). PBMCs labelled with cell trace violet (Violet Proliferation Dye 450, BD Horizon) were stimulated with 10 μM M1$_{58–66}$ peptide (GILGFVFTL), or DMSO as unstimulated control, and cultured for 10 days. On day 9, cells were tetramer stained, stained with our TAME panel (except CD71-BV421), fixed and permabilised and intracellularly stained for IFN-γ, TNF, Perforin and Granzyme B.

Statistical analyses

Unless otherwise indicated, data were analyzed using GraphPad Prism (v.10.2.2, GraphPad) and R (v4.4.0). Non-parametric two-sided Kruskal-Wallis with Dunn's correction for multiple comparisons were performed with Rstatix package (v0.7.2) (plotted with ggplot2 (10)). Two-way ANOVA with Tukey's test was used to compare proportions of A2/M1$_{58}$ memory phenotypes between age groups (test and graphing performed with GraphPad Prism). Spearman's test was used to determine significant correlations. Differences were considered significant at $p<0.05$.

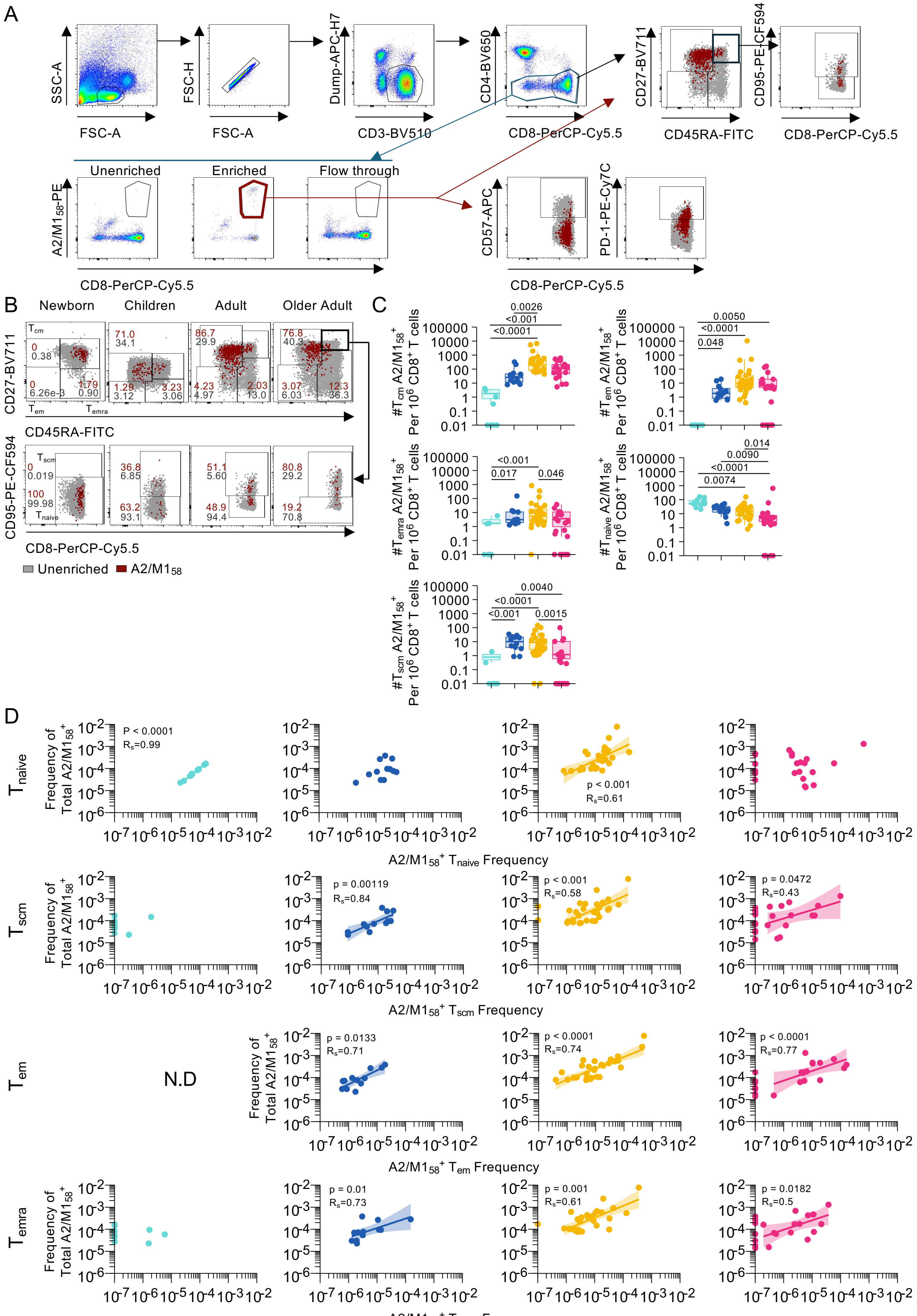

Figure S1 Menon et al

Figure S1. Gating strategy to identify A2/M1$_{58}^{+}$CD8^{+} T-cells and their memory subsets. (A) Gating strategy to identify and phenotype A2/M1$_{58}^{+}$CD8^{+} T-cells. **(B)** Representative FACS plots of A2/M1$_{58}^{+}$CD8^{+} T_{cm}-like (CD27^{+}CD45RA^{-}) cells, T_{em}-like (CD27^{-}CD45RA^{-}), T_{emra}-like (CD27^{-}CD45RA^{+}), T_{naive}-like (CD27^{+}CD45RA^{+}CD95^{-}) and T_{scm}-like (CD27^{+}CD45RA^{+}CD95^{+}) cells from all age groups. **(C)** Absolute numbers per 10^6 CD8^{+} T-cells of memory A2/M1$_{58}^{+}$ CD8^{+} T-cells per age group. Statistical significance was determined using a two-sided Kruskal–Wallis with Dunn's test for multiple comparisons. Frequencies of zero were plotted as 0.01. **(D)** Correlation of the frequency of total A2/M1$_{58}^{+}$CD8^{+} T-cells and frequency of A2/M1$_{58}^{+}$CD8^{+} T_{cm}-like cells using Spearman's rank correlation (R_s) (n=10; Newborns, n=12; Children, n=30; Adults, n=22; Older Adults)

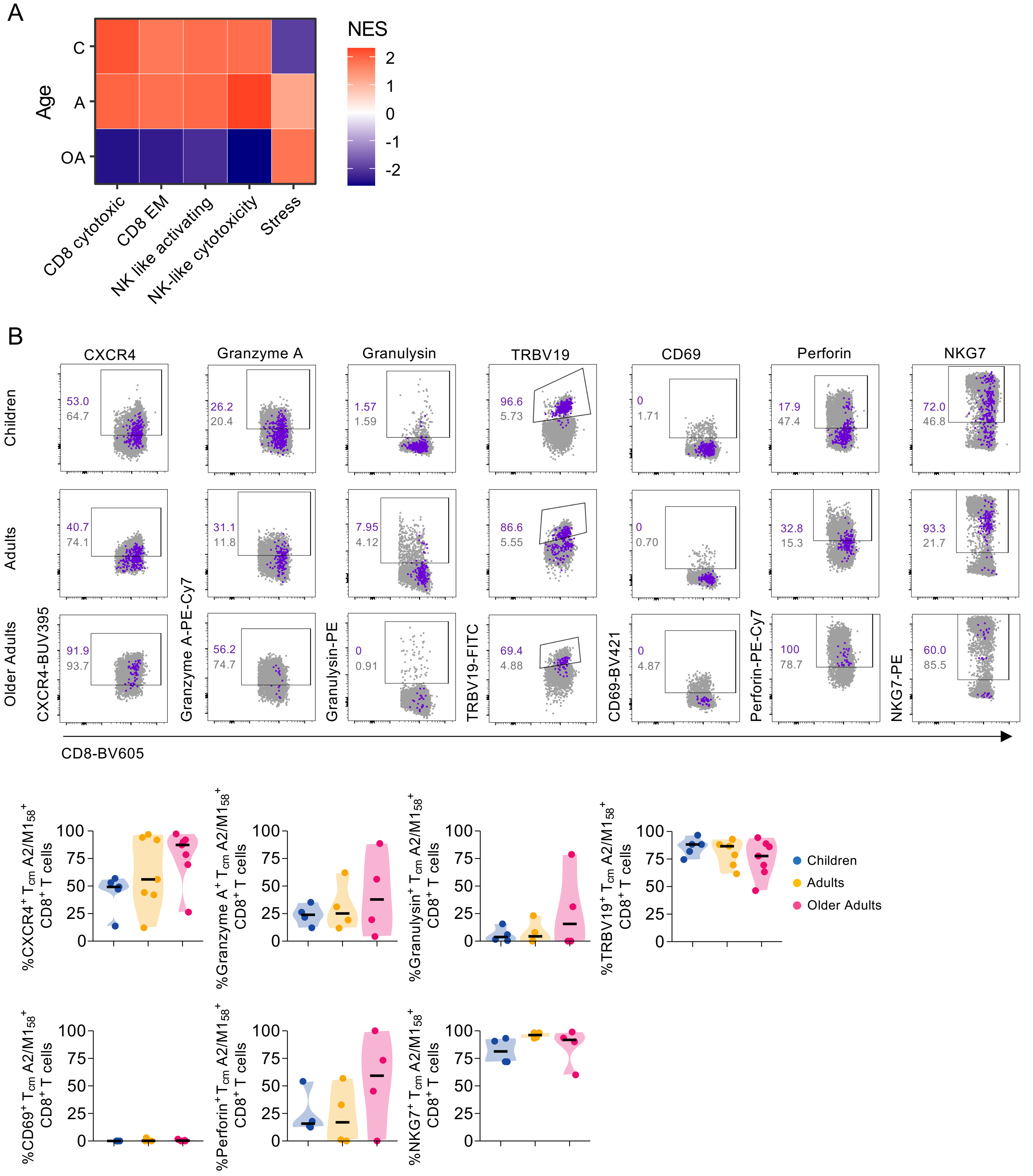

Figure S2 scRNASeq analysis and validation. (A) Heatmap of enriched pathways identified from GSEA using differentially expressed genes between each age group. All pathways shown have adjusted *p*-values < 0.05 in at least one age group. NES: Normalized enrichment score. **(B)** FACS plots of scRNASeq markers in T_{cm} A2/M1$_{58}^{+}$CD8^{+} T cells from children, adults and older adults and their expression in children (n=4-5), adults (n=4-7) and older adults (n=4-7). Bars represent median. Statistical significance was determined using a two-sided Kruskal–Wallis with Dunn's test for multiple comparisons.

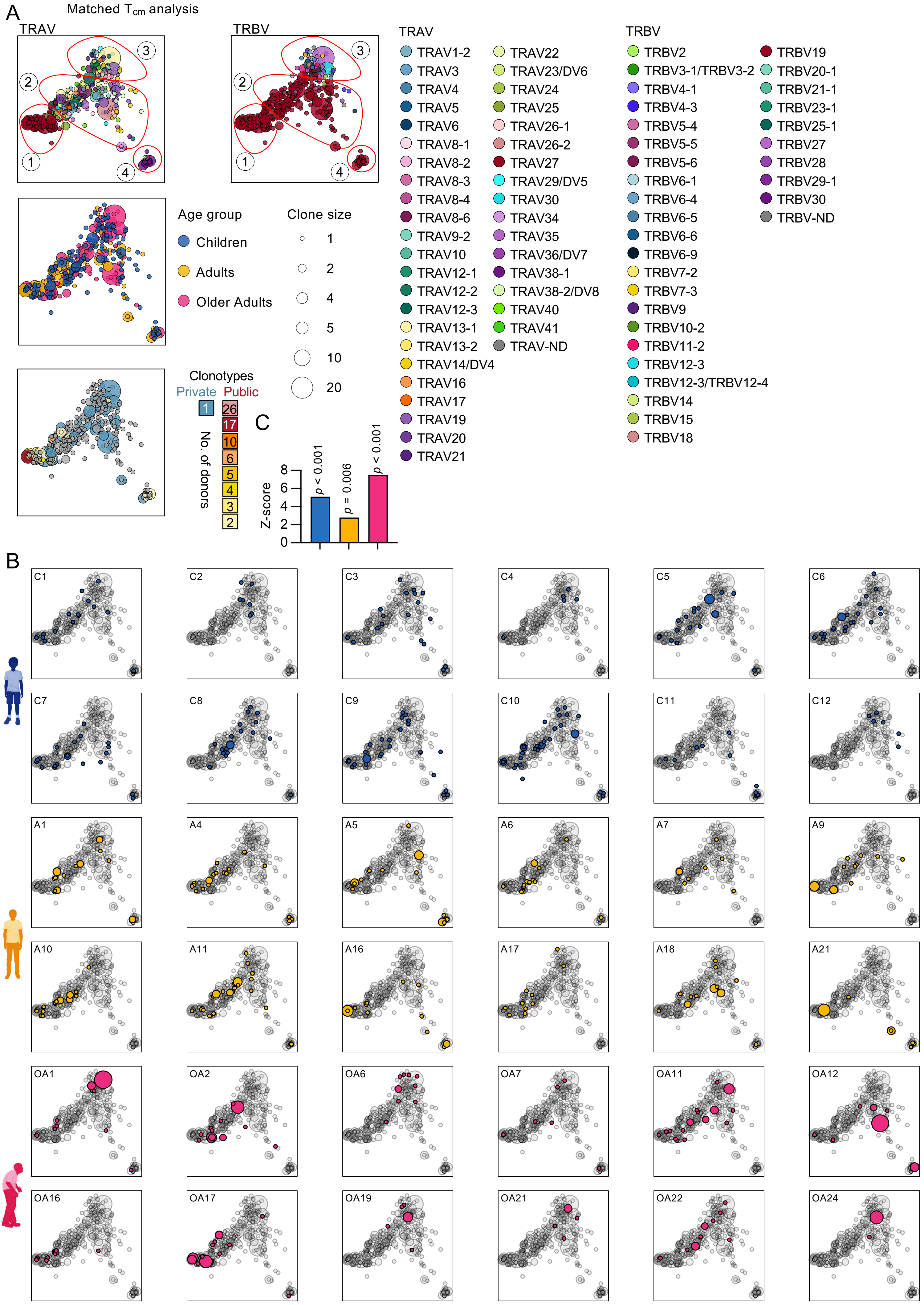

Figure S3 Menon et al

Figure S3. TCR repertoire of matched T_{cm} A2/M1$_{58}$$^+$CD8$^+$ cells across age groups. (A) A 2D kernel principal-component analysis (kPCA) projection of the total A2/M1$_{58}$$^+$CD8$^+$ T_{cm} TCR landscape for children, adults and older adults generated by TCRdist. Dots are coloured by TRAV or TRBV gene usage, age group or by public or private clonotype. Encoding clone size indicated by symbol size. **(B)** kPCA projection of individual donors, colored dots indicate clones in the respective donor, gray dots indicate the overall kPCA of all donors combined. Donor IDs are given on the top left of the plot. **(C)** Z-score for intra- versus inter-donor distance, larger Z means intra-donor distances are smaller than inter-donor distances, that is greater heterogeneity across donors.

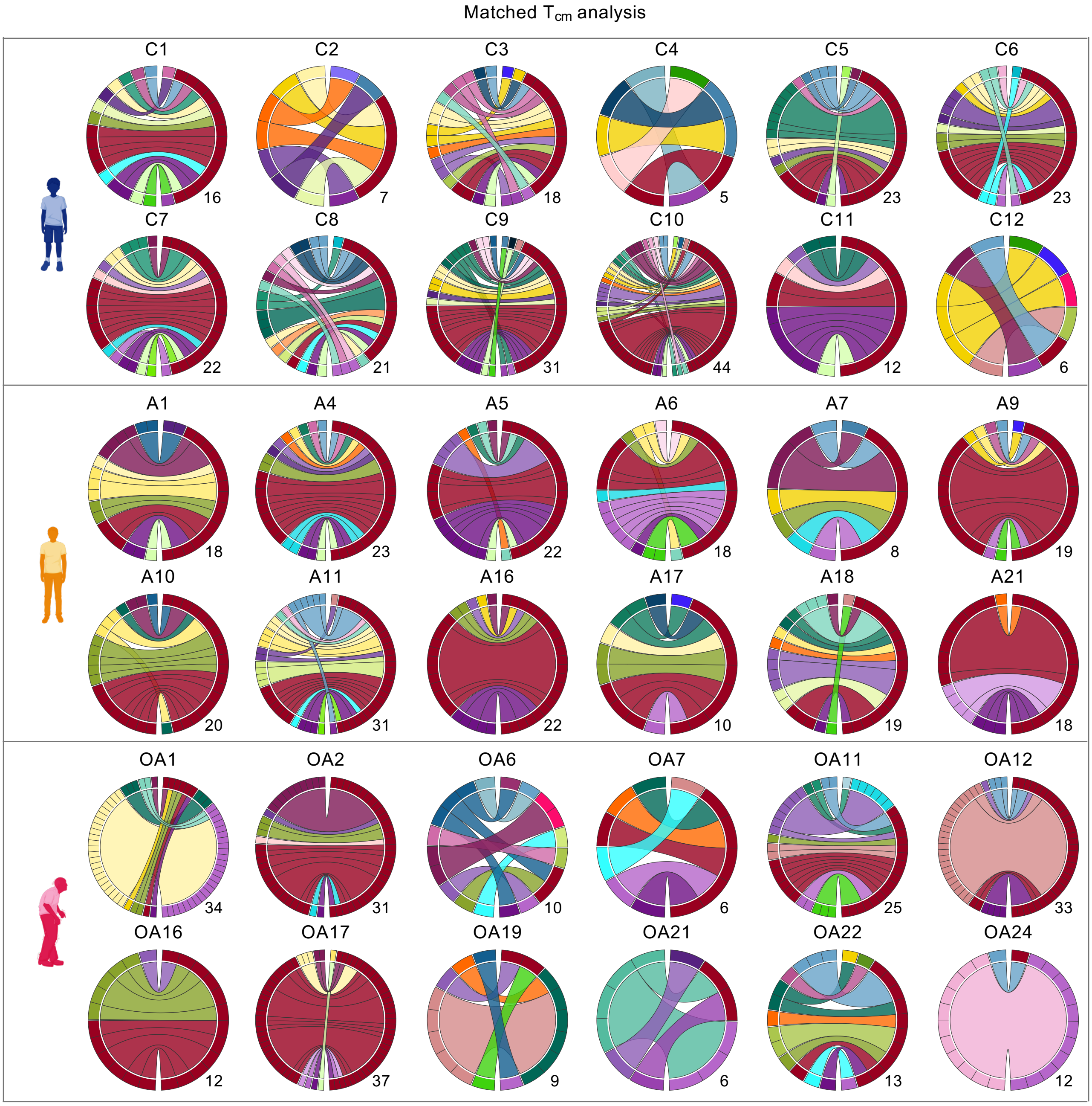

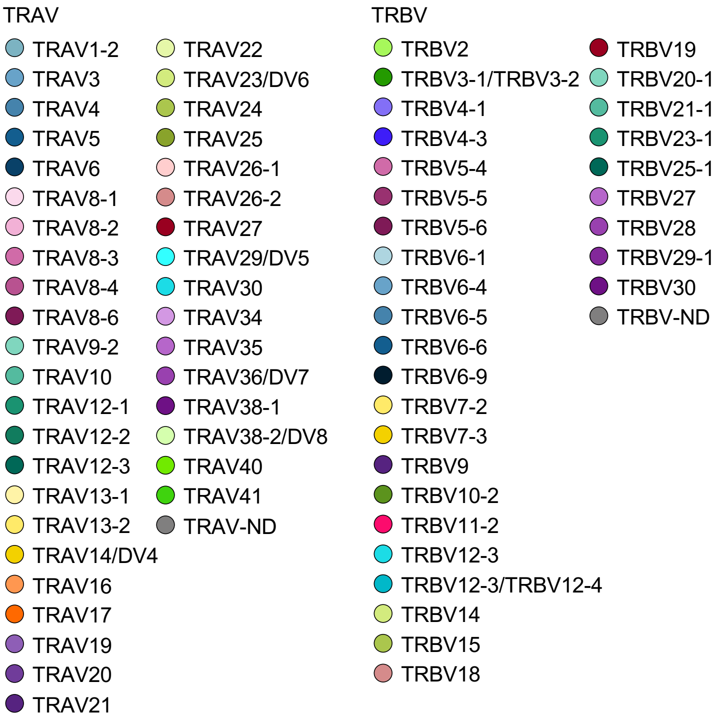

Figure S4. Circos analysis of matched T_{cm} A2/M1$_{58}$$^{+}$CD8^{+} cells in individual donors. TRAV and TRBV clonotype pairing for individual donors within each age group illustrated by circos plots. Left arch segment colors indicate TRAV usage, right outer arch colors depict TRBV usage. Connecting lines indicated TRAV-TRBV gene pairing and are colored based on their TRAV usage and segmented based on their CRD3α and CDR3β sequence, the thickness is proportional to the number of TCR clones with the respective pair. The number of sequences considered for each circos plot is shown at the right bottom.

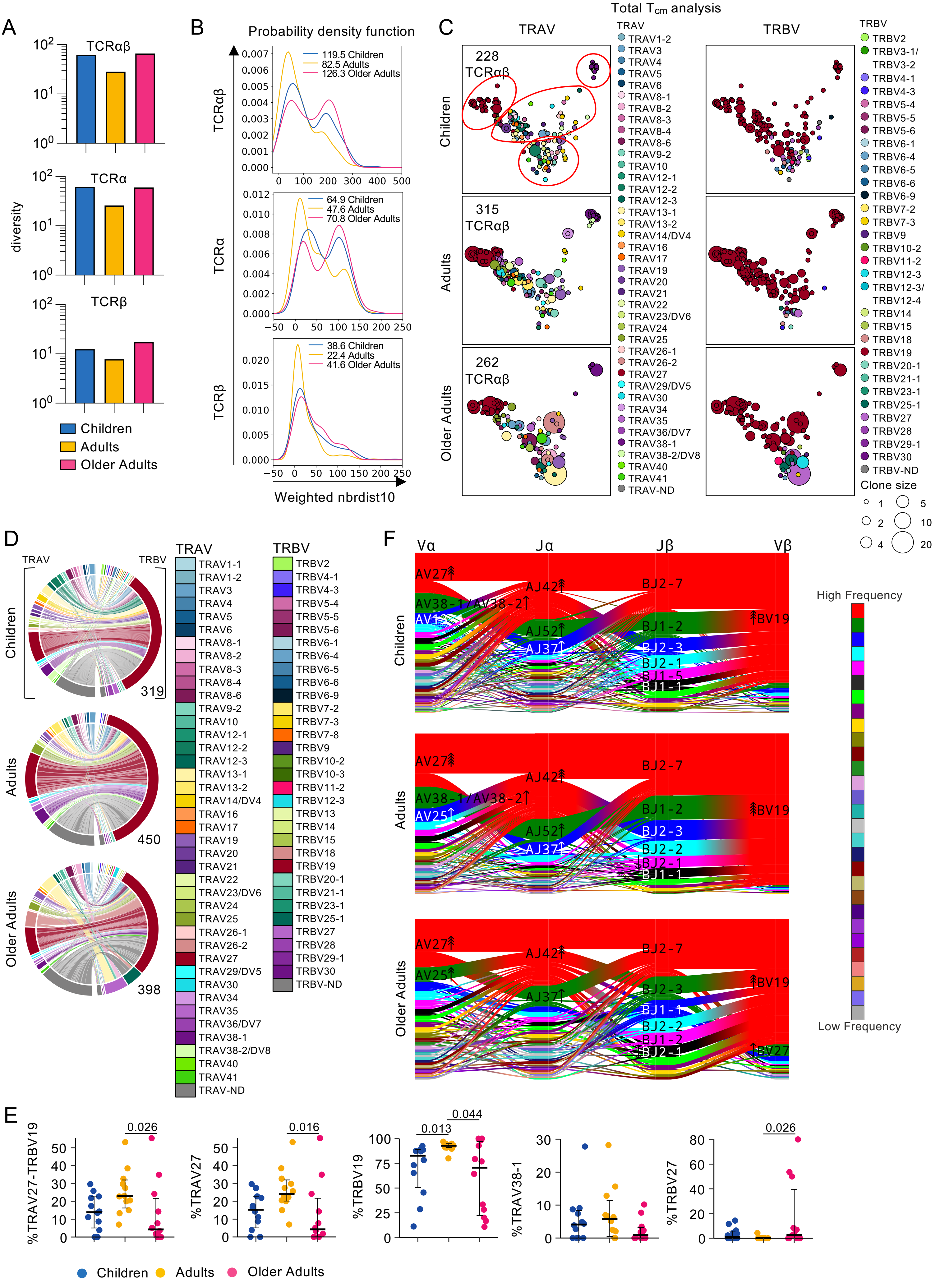

Figure S5 Menon et al

Figure S5. TCRαβ repertoire of total A2/M1$_{58}$$^{+}$CD8^{+} T$_{cm}$ across age groups. (A) TCR diversity of paired TCRαβ, TCRα and TCRβ-chains measured by TCRdiv. **(B)** Smoothed density profiles of neighbor distance distribution are shown for each age group. A lower distribution peak indicates more clustered A2/M1$_{58}$$^{+}$CD8^{+} T$_{cm}$ single paired TCRαβ, TCRα or TCRβ repertoire, average distance values for each age group are depicted within the plot. PDF, probability density function. **(C)** 2D kernel principal-component analysis (PCA) projection of the total A2/M1$_{58}$$^{+}$CD8^{+} T$_{cm}$ TCR landscape colored by TRAV or TRBV gene usage for children, adults and older adults generated by TCRdist. Encoding clone size indicated by symbol size. The number of paired TCRαβ sequences in each plot is displayed on the top left. Red circles were manually drawn to indicate regions of interest. **(D)** Circos plots of TRAV and TRBV clonotype pairing per age group. Left arch segment colors indicate TRAV usage, right outer arch colors depict TRBV usage. Connecting lines indicated *TRAV–TRBV* gene pairing and are colored based on their TRAV usage and segmented based on their CRD3α and CDR3β sequence, the thickness is proportional to the number of TCR clones with the respective pair. The number of sequences considered for each circos plot is shown at the right bottom. **(E)** Proportion of sequences expressing TRAV27-TRRBV19, TRAV27, TRBV19, TRAV38-1 or TRBV27. Bars represent median and interquartile range (IQR). Statistical significance was determined using a two-sided Kruskal–Wallis with Dunn's test for multiple comparisons. **(F)** Gene segment usage and gene-gene pairing landscapes, vertical stacks indicate V and J segments and gene pairing indicated by curved paths, thickness is proportional to the number of TCR clones within the respective gene pairing. Genes are colored by frequency within the repertoire. Up or down arrows indicate the enrichment of gene segments relative to background frequencies, with each arrowhead indicating a 2-fold enrichment.

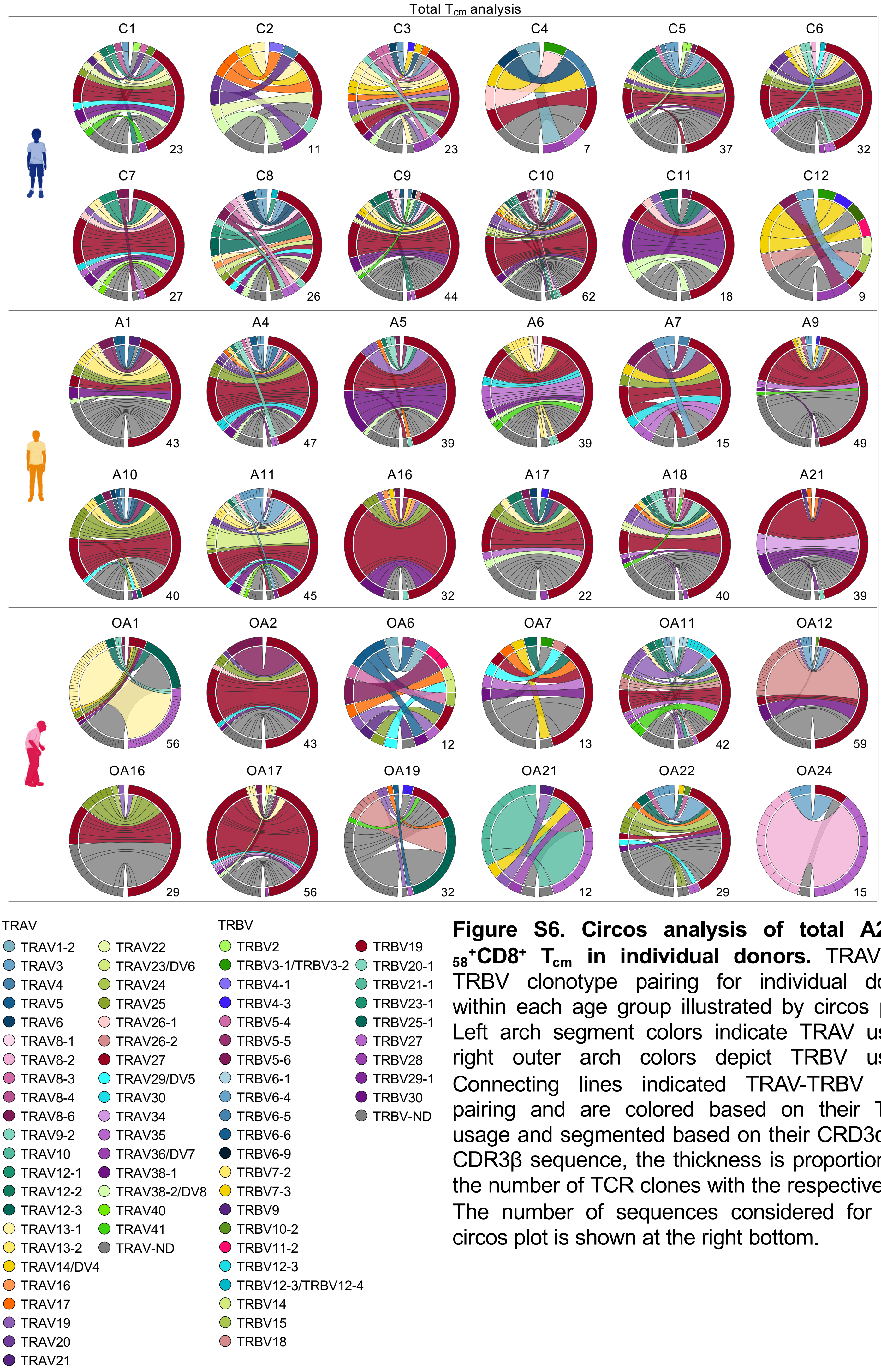

Figure S6. Circos analysis of total A2/M1-$_{58}$$^{+}$CD8^{+} T$_{cm}$ in individual donors. TRAV and TRBV clonotype pairing for individual donors within each age group illustrated by circos plots. Left arch segment colors indicate TRAV usage, right outer arch colors depict TRBV usage. Connecting lines indicated TRAV-TRBV gene pairing and are colored based on their TRAV usage and segmented based on their CRD3α and CDR3β sequence, the thickness is proportional to the number of TCR clones with the respective pair. The number of sequences considered for each circos plot is shown at the right bottom.

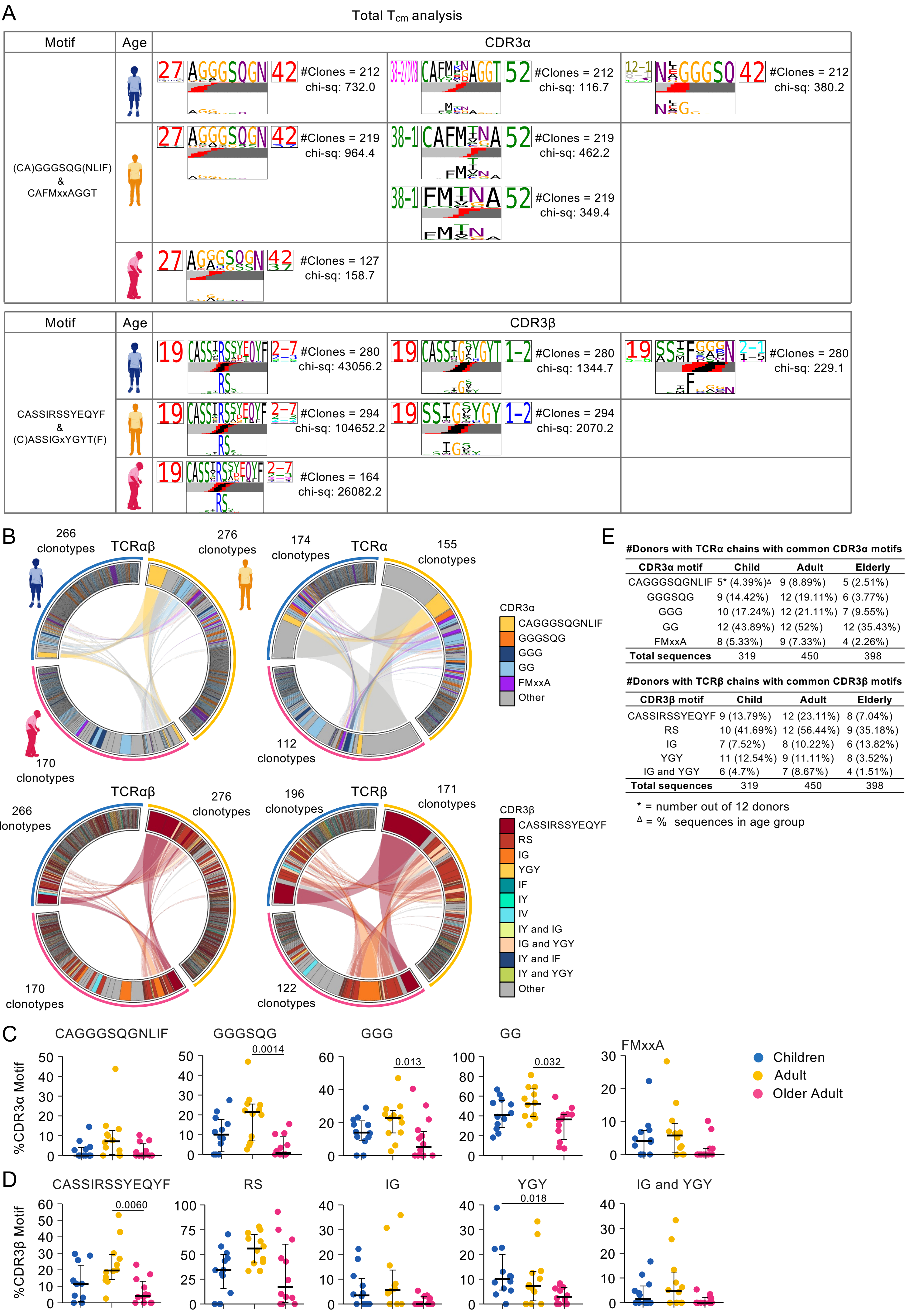

#Donors with TCRα chains with common CDR3α motifs

CDR3α motif	Child	Adult	Elderly
CAGGGSQGNLIF	5* (4.39%)Δ	9 (8.89%)	5 (2.51%)
GGGSQG	9 (14.42%)	12 (19.11%)	6 (3.77%)
GGG	10 (17.24%)	12 (21.11%)	7 (9.55%)
GG	12 (43.89%)	12 (52%)	12 (35.43%)
FMxxA	8 (5.33%)	9 (7.33%)	4 (2.26%)
**Total sequences**	319	450	398

#Donors with TCRβ chains with common CDR3β motifs

CDR3β motif	Child	Adult	Elderly
CASSIRSSYEQYF	9 (13.79%)	12 (23.11%)	8 (7.04%)
RS	10 (41.69%)	12 (56.44%)	9 (35.18%)
IG	7 (7.52%)	8 (10.22%)	6 (13.82%)
YGY	11 (12.54%)	9 (11.11%)	8 (3.52%)
IG and YGY	6 (4.7%)	7 (8.67%)	4 (1.51%)
**Total sequences**	319	450	398

* = number out of 12 donors
Δ = % sequences in age group

Figure S7 Menon et al

Figure S7. CDR3αβ motifs of total A2/M1$_{58}$$^{+}$CD8^{+} T$_{cm}$ across age groups. (A) The top-scoring A2/M1$_{58}$$^{+}$CD8^{+} T$_{cm}$ CDR3α (left TCR logo) and CDR3β (right TCR logo) sequence motifs for each age group. Each logo depicts the V (left side) and J (right side) gene frequencies with the CDR3 amino acid sequence in the middle with the full height (top) and scaled (bottom) by per-residue reparative entropy to background frequencies derived from TCRs with matching gene-segment composition to highlight motif positions under selection. The middle section indicates the inferred rearrangement structure by source region (light grey for V-region, dark grey for J, black for D and red for N insertions) of the grouped receptors. **(B)** Frequency of common CDR3αβ motifs within total A2/M1$_{58}$$^{+}$CD8^{+} T$_{cm}$ across age groups. Connecting lines represent TCRα, TCRβ or paired TCRαβ-chains shared between each age group. Plots are coloured by CDR3α motif (top row) or CDR3β (bottom row). **(C)** Proportion of common CDR3α motifs or **(D)** CDR3β motifs across children, adults and older adults. Statistical significance was determined using a two-sided Kruskal–Wallis with Dunn's test for multiple comparisons. **(E)** Table of the number of donors in each age group expressing common CDR3αβ motifs. Percentage of sequences expressing these motifs in each age group are in parentheses.

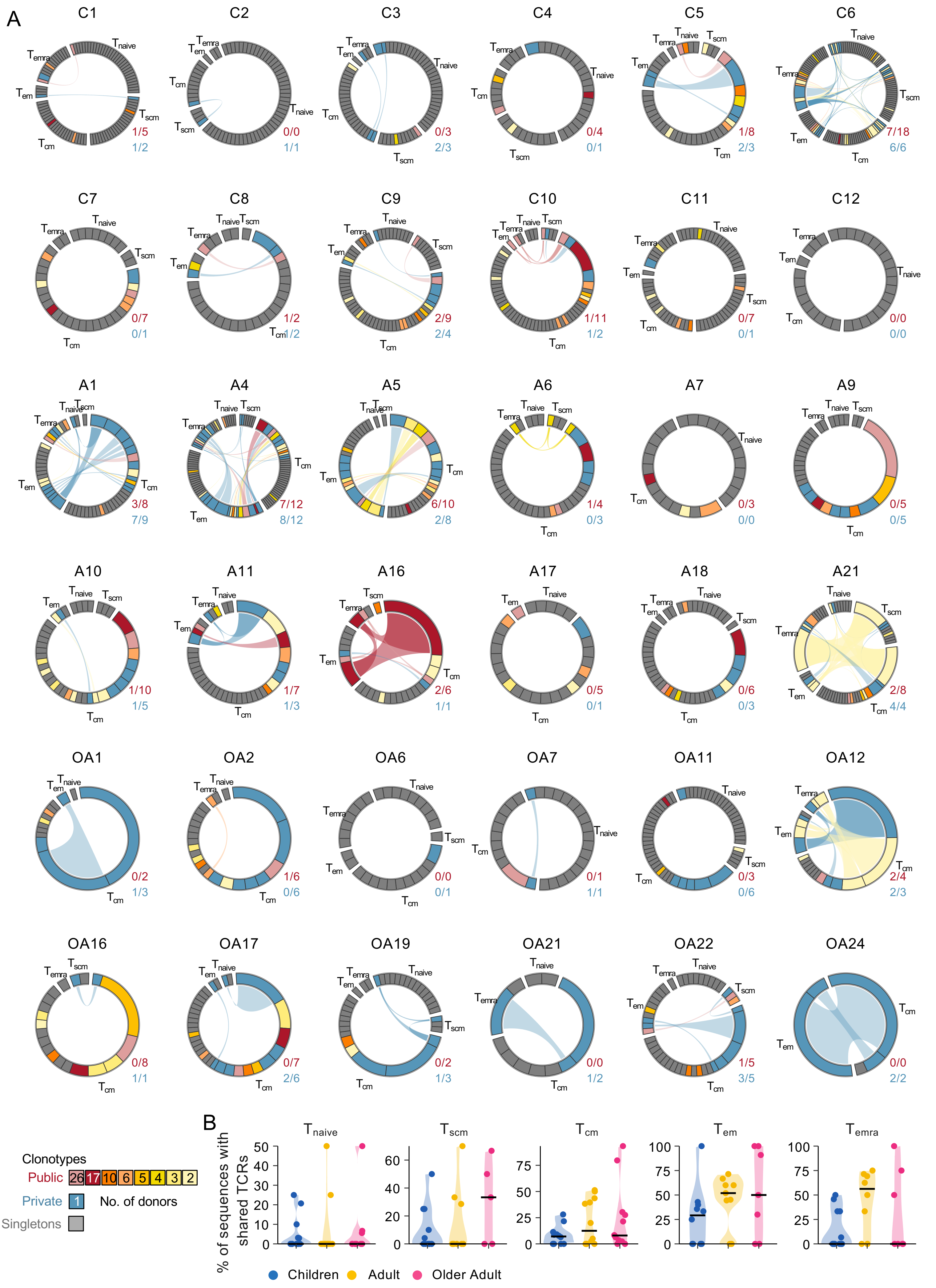

Figure S8 Menon et al

Figure S8. Sharing of public and private clonotypes between phenotype subsets across individual participants. (A) Frequency of high-prevalent (≥2 similar TCRs within a single individual) public (shared) and private (not shared) clonotypes across different phenotypes per age group. Connecting lines represent paired TCRαβ-chains shared between each memory subset. Plots are coloured by whether clonotypes are public (red, orange or yellow) or private (blue). Dark red represents high-prevalent public TCR (TRAV27, TRAJ42, CDR3α GAGGGSQGNLIF, TRBV19, TRBV2–7 and CDR3β CASSIRSSYEQYF), whereas the light red are clonotypes expressing the full public TCRβ chain (TRBV19, TRBV2–7 and CDR3β CASSIRSSYEQYF) but the TCRα-chain could not be identified. Each plot represents an individual donor. The fraction of public (red) or private (blue) TCRαβ clonotypes shared across phenotypes out of the total number of public or private TCRαβ clonotypes are shown on the bottom right. **(B)** Proportion of TCRs shared with another age group subset across phenotypes. Statistical significance was determined using a two-sided Kruskal–Wallis with Dunn’s test for multiple comparisons.

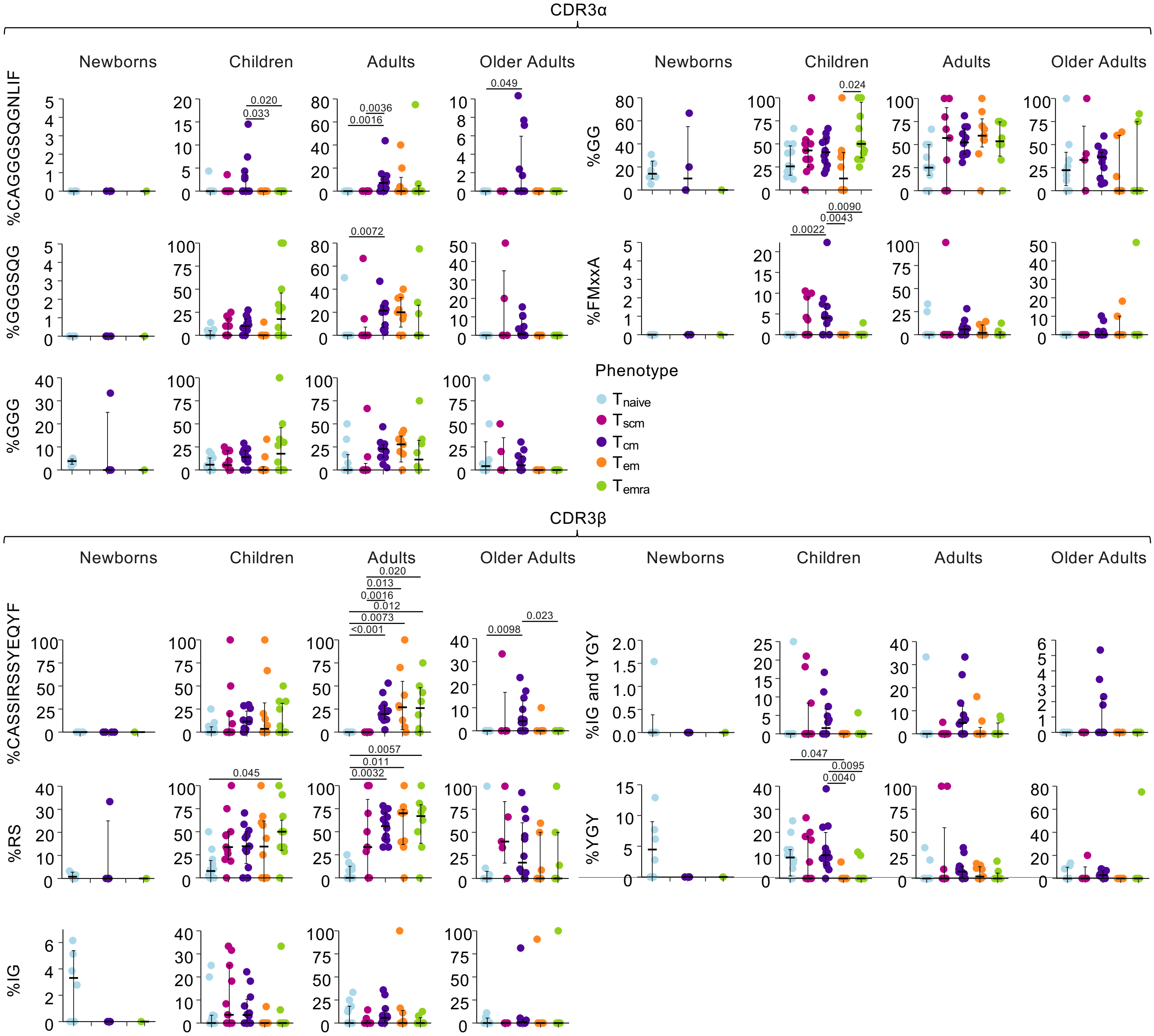

Figure S9. Frequency of common CDR3αβ motifs in each phenotype subset across age groups. Proportion of sequences containing common CDR3αβ motifs in each phenotype subset per age group.

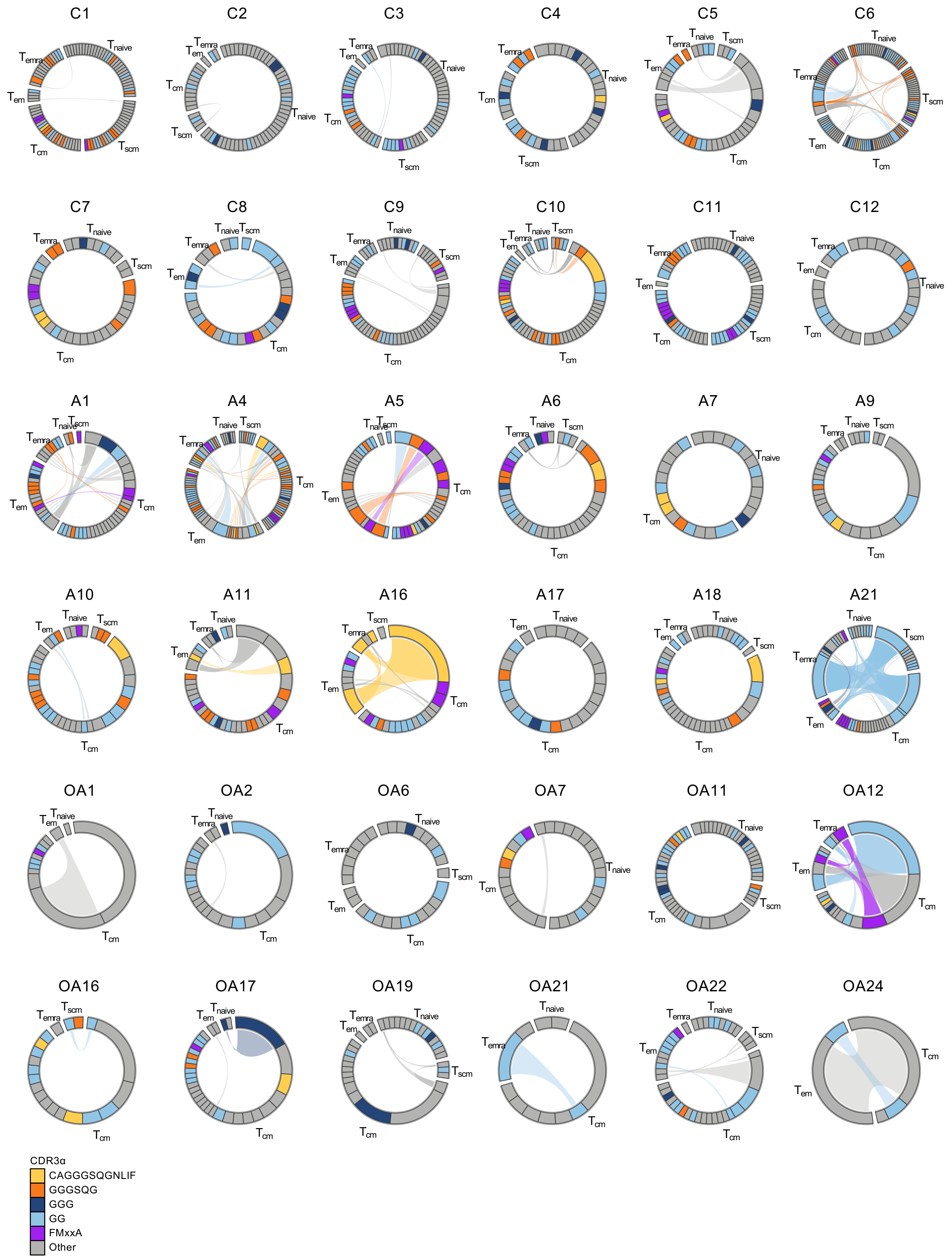

Figure S10. Frequency of common CDR3α motifs across phenotype subsets per individual participant. Connecting lines represent paired TCRαβ-chains shared between each memory subset. Plots are coloured by CDR3α motif. Each plot represents an individual.

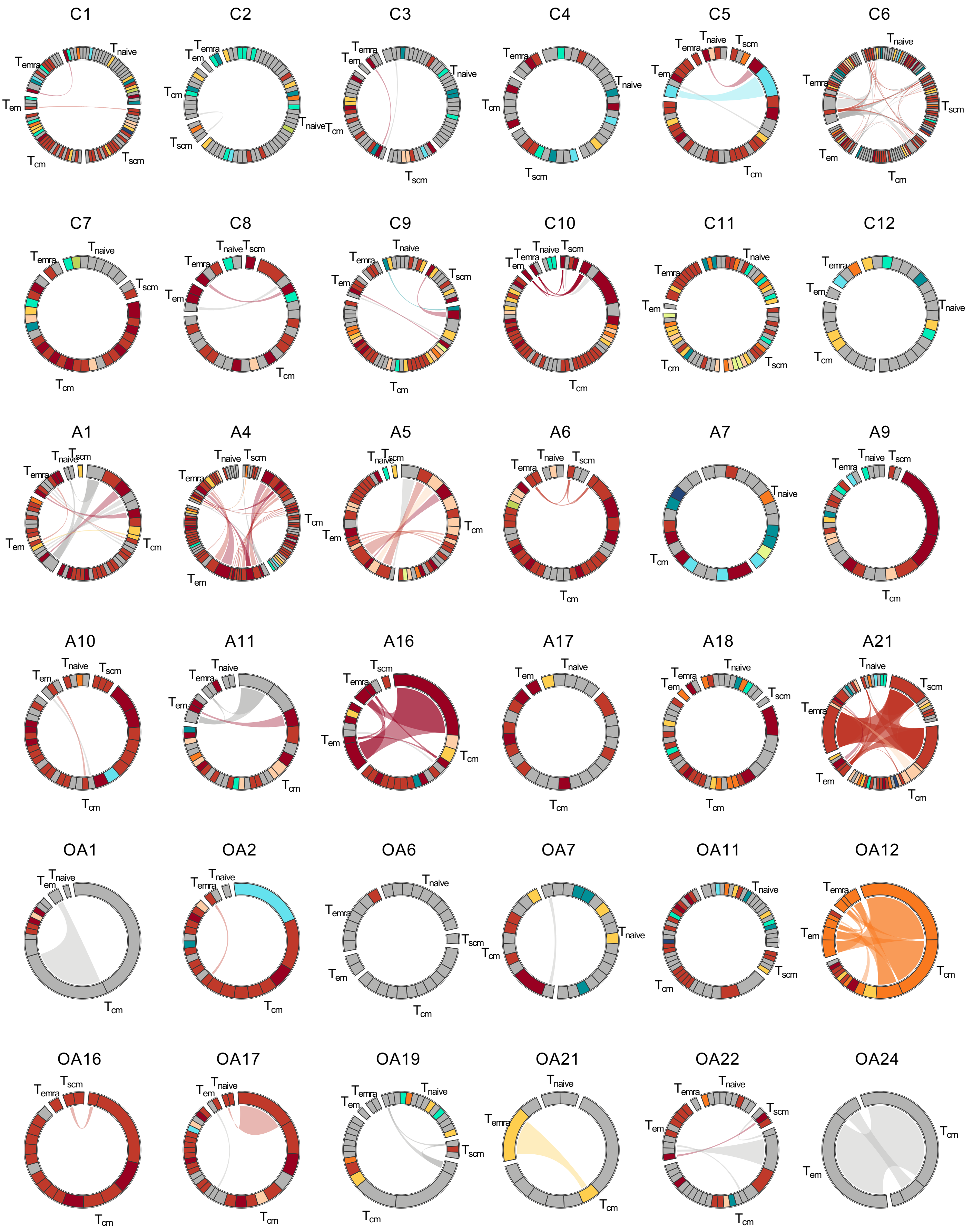

Figure S11. Frequency of common CDR3β motifs across phenotype subsets per individual participant. Connecting lines represent paired TCRαβ-chains shared between each memory subset. Plots are coloured by CDR3β motif. Each plot represents an individual.

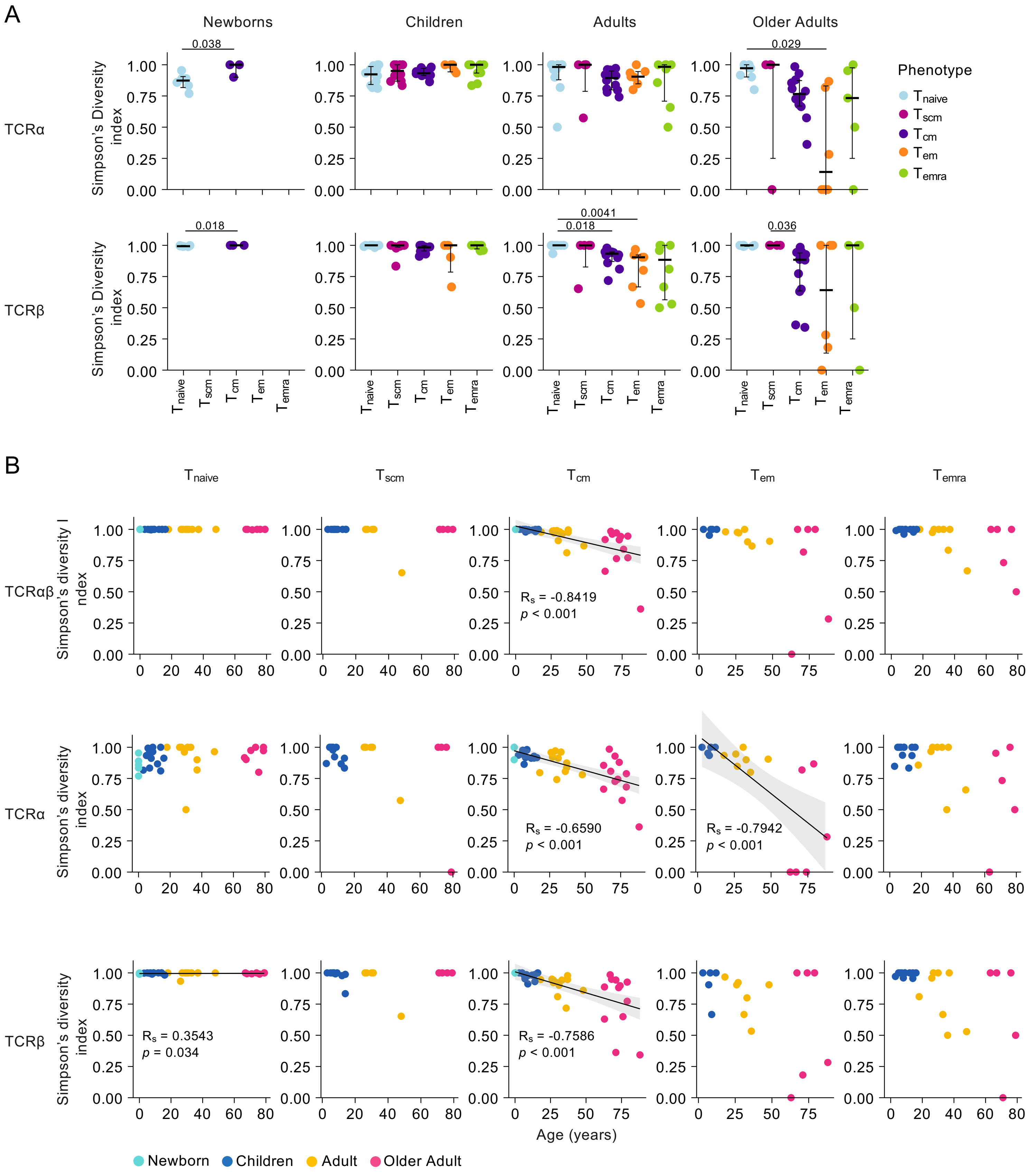

Figure S12. TCR diversity across age groups. (A) SDI of single TCRα- or TCRβ-chains comparing phenotype subsets within each age group. Statistical significance was determined using a two-sided Kruskal–Wallis with Dunn's test for multiple comparisons. **(B)** SDI of single TCRα- or TCRβ-chains versus age in each memory subset per age group.

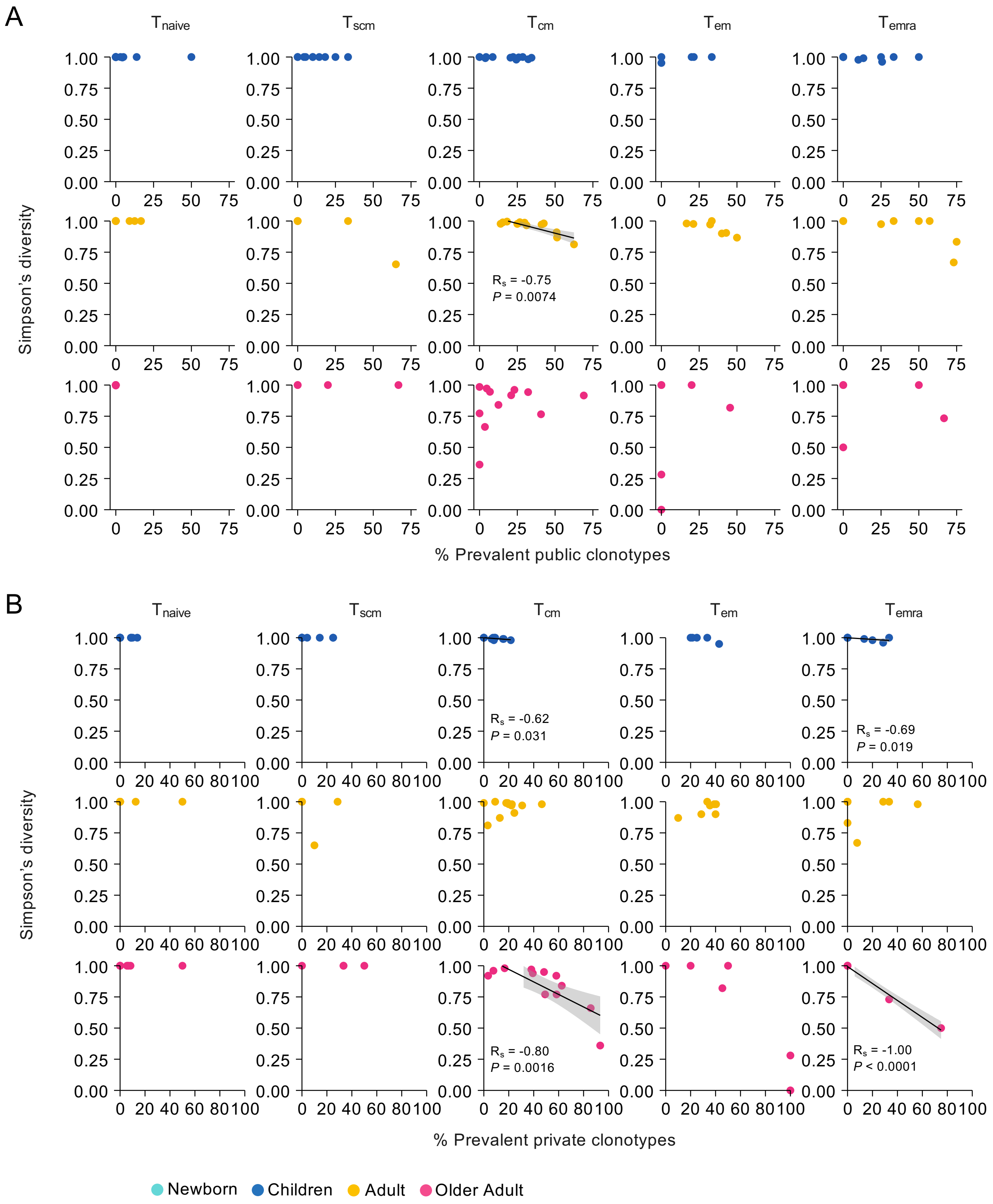

Figure S13. TCR diversity across age groups in each memory T-cell subset. (A) SDI of paired TCRαβ-chains versus age in each memory subset per age group. **(B-C)** SDI of paired TCRαβ-chains versus proportion of (B) public clonotypes or (C) private clonotypes within A2/M1$_{58}$$^{+}$CD8^{+} T-cell memory subsets. Significant correlations were determined using Spearman's rank correlation (R_s).

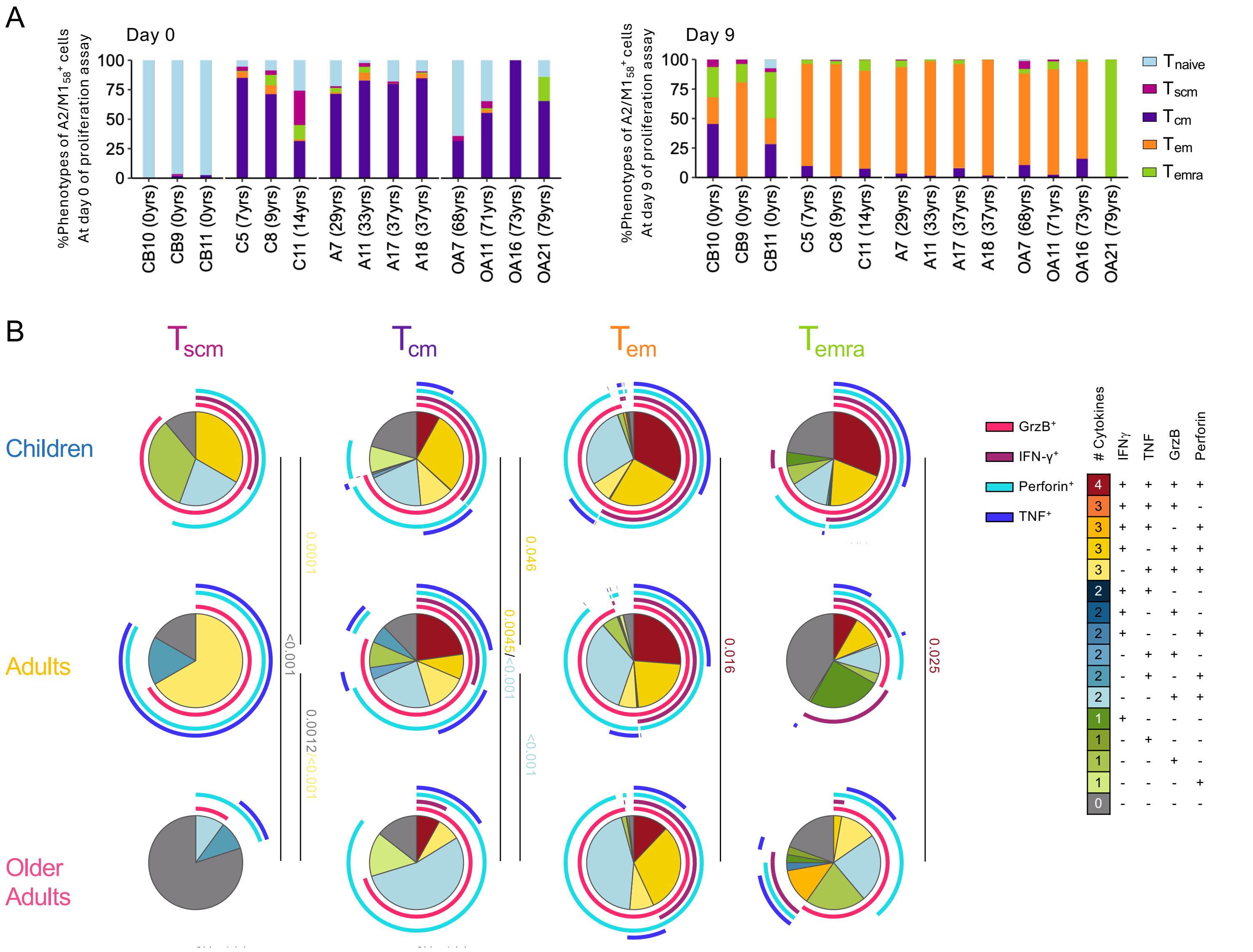

Figure S14. Polyfunctionality of T_{cm} and T_{em} A2/M1$_{58}$$^+$CD8$^+$ T-cell. (A) Phenotypic distribution of individual donors at the start of the proliferation assay (day 0; left plot) or day 9 (right plot; divided A2/M1$_{58}$$^+$CD8$^+$ T-cells). N=3 children and n=4 adults and older adults **(B)** Pie charts representing average fractions of divided memory A2/M1$_{58}$$^+$CD8$^+$ T cells, the number of co-expressed molecules IFN-γ, TNF, GrzB and perforin (slices) and specific combination (arcs). Statistical analysis was performed using a two-sided Tukey's multiple comparisons test. Exact significant *P* values are indicated in similar colors as the representative slice.

Table S1 Demographics of healthy HLA-A*02:01-expressing participants in our study

Cohort	ID	Age group	Age (years)	Sex	HLA-A		HLA-B		HLA-C		TAME	Ex vivo TCR	Proliferation	scRNASeq	scRNASeq validation
Newborn	CB1	Newborn	0	Unk	02:01	24:02	35:03	50:01	06:02	12:03	Yes				
Median 0 yrs	CB2	Newborn	0	Unk	01:01	02:01	41:01	51:01	02:02	07:01	Yes[a]				
Range 0	CB3	Newborn	0	F	01:01	02:01	08:01	41:01	02:08	07:01	Yes				
27.3% F	CB4	Newborn	0	M	01:01	02:01	08:01	41:01	02:08	07:01	Yes				
5 Unk sex	CB5	Newborn	0	Unk	02:01	32:01	44:02	51:01	02:02	05:01	Yes				
	CB6	Newborn	0	Unk	02:01	03:01	07:02	15:01	03:03	07:02	Yes	Yes		Yes	
	CB7	Newborn	0	Unk	02:01	68:02	14:02	51:01	01:02	08:02	Yes	Yes		Yes	
	CB8	Newborn	0	M	02:01	11:01	14:01	35:01	04:01	08:02	Yes	Yes			
	CB9	Newborn	0	M	02:01	03:01	07:02		07:02		Yes	Yes	Yes	Yes	
	CB10	Newborn	0	F	02:01	33:03	07:02	58:01	03:02	07:02	Yes	Yes	Yes		
	CB11	Newborn	0	F	02:01		44:02		05:01	07:04	Yes	Yes	Yes		
Children	C1	Children	3	M	02:01	29:02	44:03	51:01	14:02	16:01	Yes	Yes			
Median 8 yrs	C2	Children	5	F	01:01	02:01	08:01	40:01	03:03	07:01	Yes	Yes			
Range 3-16 yrs	C3	Children	6	M	02:01	32:03	15:01	44:02	04:01	05:01	Yes	Yes		Yes	
52.9% F	**SC1**	Children	6	M	02:01	11:01	15:01	35:01	03:04	04:01					Yes
	C4	Children	7	M	02:01		44:02		05:01	07:04	Yes	Yes			
	C5	Children	7	F	01:01	02:01	07:02	37:01	06:02	07:02	Yes	Yes	Yes		
	**SC2**	Children	7	F	02:01	03:01	07:02	40:01	03:04	07:02					Yes
	**SC3**	Children	7	M	02:01	24:02	07:02	08:01	07:01	07:02					Yes
	C6	Children	8	F	02:01		08:01	15:01	04:01	07:01	Yes	Yes			
	C7	Children	9	F	01:01	02:01	08:01	44:02	05:01	07:01	Yes	Yes			
	C8	Children	9	M	02:01	29:02	44:02	44:03	05:01	16:01	Yes	Yes	Yes		
	**SC4**	Children	9	F	02:01	25:01	15:01	18:01	03:03	12:03					Yes
	**SC5**	Children	12	M	02:01	24:02	07:02	44:02	05:01	07:02					Yes
	C9	Children	12	M	02:01	02:05	49:01		07:01		Yes	Yes		Yes	
	C10	Children	14	F	02:01		41:02	44:02	05:01	17:03	Yes	Yes		Yes	
	C11	Children	14	F	02:01	03:01	14:02	44:02	05:01	08:02	Yes	Yes	Yes		
	C12	Children	16	F	02:01	11:01	15:01	40:01	03:03	03:04	Yes	Yes			
Adults	A1	Adults	18	M	02:01	03:01	07:02	38:01	07:02	12:03	Yes	Yes			
Median 36 yrs	**SA1**	Adults	22	F	02:01	24:02	13:01	41:01	03:04	17:01					Yes
Range 18-58 yrs	A2	Adults	24	F	02:01	03:01	15:01	44:02	03:03	05:01	Yes				Yes
45.5% F	A3	Adults	24	F	02:01		27:05	44:03	01:02	16:01	Yes				
1 Unk sex	A4	Adults	26	M	02:01	11:01	51:01	55:01	02:02	03:03	Yes				
	**A5**	Adults	27	F	02:01	24:02	18:01	44:03	04:01	07:01	Yes	Yes			Yes
	**A6**	Adults	27	M	02:01	02:01	15:01	44:03	03:03	16:01	Yes	Yes			
	A7	Adults	29	F	02:01	30:01	44:02	58:01	05:01	07:18	Yes	Yes	Yes		
	A8	Adults	29	F	02:01		15:22	18:01	07:01	12:03	Yes				
	**A9**	Adults	30	F	02:01	25:01	18:01	44:02	05:01		Yes	Yes			
	**A10**	Adults	31	M	02:01		40:01	51:01	03:04	14:02	Yes	Yes			
	**SA2**	Adults		M	02:01	03:01	07:02	27:05	02:02	07:02					Yes
	A11	Adults	33	F	02:01	11:01	35:01	39:01	Unk		Yes	Yes	Yes	Yes	
	**A12**	Adults	33	F	01:01	02:01	15:01	50:01	03:03	06:02	Yes				Yes
	A13	Adults	34	M	02:01	26:01	38:01	55:01	03:02	12:03	Yes				
	**A14**	Adults	35	F	02:01	26:01	27:05	44:02	01:02	05:01	Yes				

	A15	Adults	36	M	01:01	02:01	07:02	37:01	06:02	07:02	Yes				
	A16	Adults	36	M	02:01	11:01	35:01	39:01	Unk		Yes	Yes			
	A17	Adults	37	F	01:01	02:01	07:02	57:01	06:02	07:02	Yes	Yes	Yes		
	A18	Adults	37	M	01:01	02:01	07:02	44:02	05:01	44:02	Yes	Yes	Yes	Yes	
	**A19**	Adults	39	M	02:01	03:01	44:05	56:01	02:02	07:02	Yes				
	**A20**	Adults	45	M	02:01	03:01	07:02	51:01	07:02	15:02	Yes				Yes
	A21	Adults	48	F	02:01	03:02	18:01	35:08	Unk		Yes	Yes			
	A22	Adults	50	F	02:01	03:01	15:01	47:01	03:03	06:02	Yes				
	A23	Adults	51	Unk	02:01	11:01	07:02	44:02	Unk		Yes				
	A24	Adults	51	M	02:01		07:02	39:01	07:02	13:03	Yes	Yes		Yes	
	**A25**	Adults	53	M	02:01	02:01	27:05	35:02	04:01	15:11	Yes				
	**A26**	Adults	54	F	02:01	24:02	07:02	15:24	03:03	07:02	Yes				
	**SA3**	Adults	56	M	02:01	07:02	44:03	07:02	16:01						Yes
	A27	Adults	57	M	02:01	03:01	07:02	40:01	03:04	07:02	Yes				
	A28	Adults	57	F	02:01	32:01	35:03	40:02	04:01	15:02	Yes				
	A29	Adults	57	M	01:01	02:01	18:01	35:01	04:01	07:01	Yes				
	A30	Adults	58	M	02:01	32:01	40:01	44:02	03:04	05:01	Yes				
Older Adults	OA1	Older Adults	63	M	02:01	03:01	07:02	50:01	06:02	07:02	Yes	Yes			
Median 72 yrs	**OA2**	Older Adults	63	F	02:01	03:01	07:02		07:02	07:02	Yes	Yes			
Range 63-88	OA3	Older Adults	65	Unk	02:01	30:01	13:01	44:03	06:02	16:01	Yes				
63.0% F	OA4	Older Adults	65	F	02:01		15:01	27:05	03:04	06:02	Yes				
4 Unk sex	**OA5**	Older Adults	65	F	02:01	33:01	39:01	41:01	02:02	12:03	Yes				
	OA6	Older Adults	67	Unk	01:01	02:01	13:02	44:32	Unk		Yes	Yes			
	OA7	Older Adults	68	F	02:01	30:01	18:01	44:02	05:01	07:01	Yes	Yes	Yes		
	OA8	Older Adults	68	Unk	01:01	02:01	08:01	14:01	07:01	08:02	Yes				
	**OA9**	Older Adults	68	F	02:01	24:02	14:02	57:01	07:01	08:02	Yes				Yes
	OA10	Older Adults	70	F	02:01	24:02	27:05	40:01	Unk		Yes[a]				
	**SOA1**	Older Adults	71	M	01:01	02:01	27:05	44:02	01:02	05:01					Yes
	OA11	Older Adults	71	F	02:01		15:01	44:02	Unk		Yes	Yes	Yes	Yes	
	**OA12**	Older Adults	71	F	02:01	03:01	35:03	44:02	04:01	05:01	Yes	Yes			Yes
	OA13	Older Adults	72	M	02:01	03:01	35:01	40:02	02:02	04:01	Yes				
	OA14	Older Adults	72	Unk	02:01	11:01	07:02	44:02	05:01	07:02	Yes				
	**OA15**	Older Adults	72	M	02:01	29:02	44:02	44:03	05:01	16:01	Yes				Yes
	**SOA2**	Older Adults	73	F	02:01	11:01	39:06:00	55:01	03:03	07:02					Yes
	OA16	Older Adults	73	F	02:01	11:01	40:01		03:04		Yes	Yes	Yes		
	OA17	Older Adults	74	F	02:01	24:02	35:03	44:03	04:01	12:03	Yes	Yes			
	**OA18**	Older Adults	74	F	Unk	Unk	Unk	Unk	Unk	Unk	Yes				Yes
	OA19	Older Adults	76	F	01:01	02:01	07:02	08:01	07:01	07:02	Yes	Yes			
	OA20	Older Adults	77	F	02:01	03:01	15:01	35:03	03:04	12:03	Yes				
	**SOA3**	Older Adults	79	M	02:01	11:01	44:02:00	44:02	05:01	07:02					Yes
	OA21	Older Adults	79	F	02:01		07:02	44:02	05:01	07:02	Yes	Yes	Yes		
	OA22	Older Adults	79	F	01:01	02:01	08:01	14:02	07:01	08:02	Yes	Yes		Yes	
	OA23	Older Adults	86	M	02:01	03:01	27:05	44:02	Unk		Yes[a]				
	OA24	Older Adults	88	F	01:01	02:01	08:01	13:02	Unk		Yes	Yes		Yes	

Unk=Unknown

[a]Total of <10 counted A2/M1$_{58\text{-}66}$$^{+}$CD8^{+} T cells within whole enrich fraction, sufficient for analysis of frequencies but not phenotypes

Newly recruited donors are indicated in bold, other donors were recruited as part of previous study (1).

Table S2 Total number of T_{cm} A2/$M1_{58}$$^{+}$CD8^{+} TCRs

Age group	#Paired αβ sequences	#Single TCRα chains	#Single TCRβ chains
Newborns	9	0	3
Children	228	10	81
Adults	315[a]	12	123
Older Adults	262[b]	10	126
**Total**	814	32	333

[a,b]Down sampled dataset shown in Table S4 from full dataset in Table S5.

References

1. C. E. van de Sandt *et al.*, Newborn and child-like molecular signatures in older adults stem from TCR shifts across human lifespan. *Nat. Immunol.* **24**, 1890-1907 (2023).
2. T. Menon *et al.*, CD8+ T-cell responses towards conserved influenza B virus epitopes across anatomical sites and age. *Nature Communications* **15**, 3387 (2024).
3. L. C. Rowntree *et al.*, SARS-CoV-2-specific T cell memory with common TCRαβ motifs is established in unvaccinated children who seroconvert after infection. *Immunity* **55**, 1299-1315.e1294 (2022).
4. C. E. van de Sandt *et al.*, Challenging immunodominance of influenza-specific CD8+ T cell responses restricted by the risk-associated HLA-A*68:01 allomorph. *Nature Communications* **10**, 5579 (2019).
5. C. Alanio, F. Lemaitre, H. K. Law, M. Hasan, M. L. Albert, Enumeration of human antigen-specific naive CD8+ T cells reveals conserved precursor frequencies. *Blood* **115**, 3718-3725 (2010).
6. C. E. van de Sandt *et al.*, Newborn and child-like molecular signatures in older adults stem from TCR shifts across human lifespan. *Nat Immunol* **24**, 1890-1907 (2023).
7. P. Dash *et al.*, Quantifiable predictive features define epitope-specific T cell receptor repertoires. *Nature* **547**, 89-93 (2017).
8. Z. Gu, L. Gu, R. Eils, M. Schlesner, B. Brors, circlize implements and enhances circular visualization in R. *Bioinformatics* **30**, 2811-2812 (2014).
9. H. Wickham, R. François, L. Henry, K. Müller, D. Vaughan, *dplyr: A Grammar of Data Manipulation* (2023).
10. H. Wickham, *ggplot2: Elegant Graphics for Data Analysis*, Applied Spatial Data Analysis R (Springer-Verlag New York, 2009).
